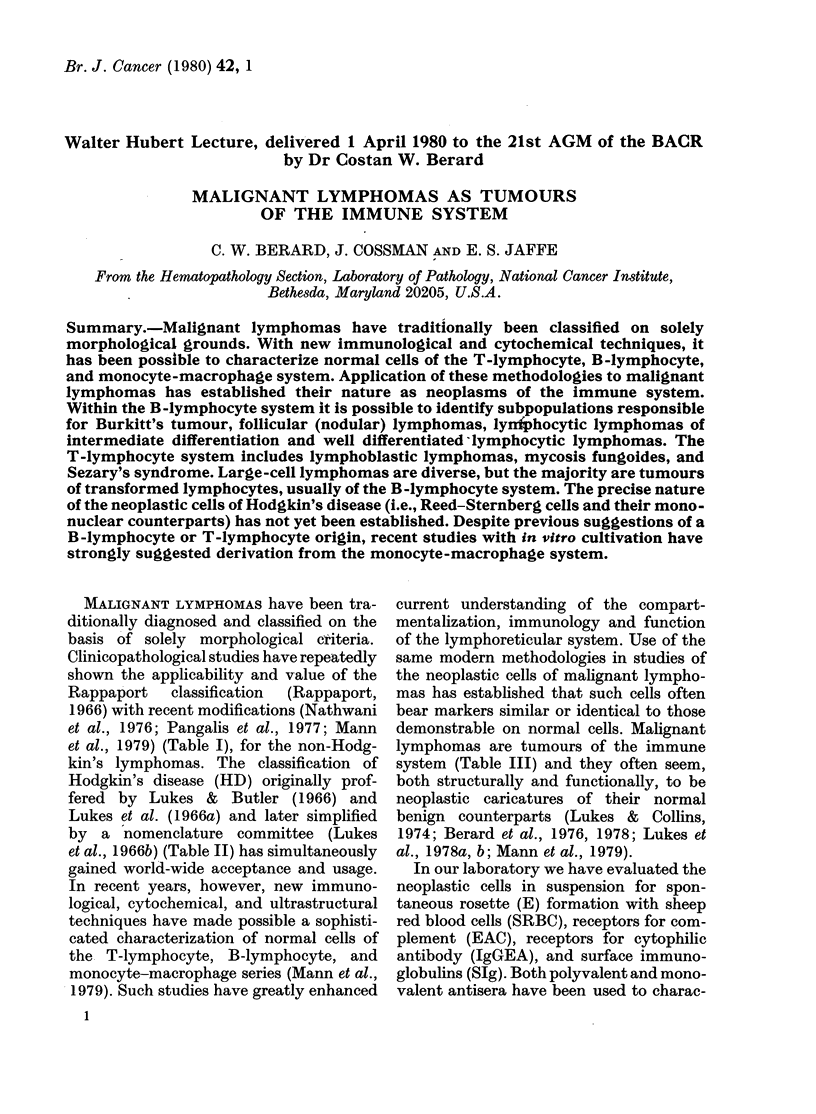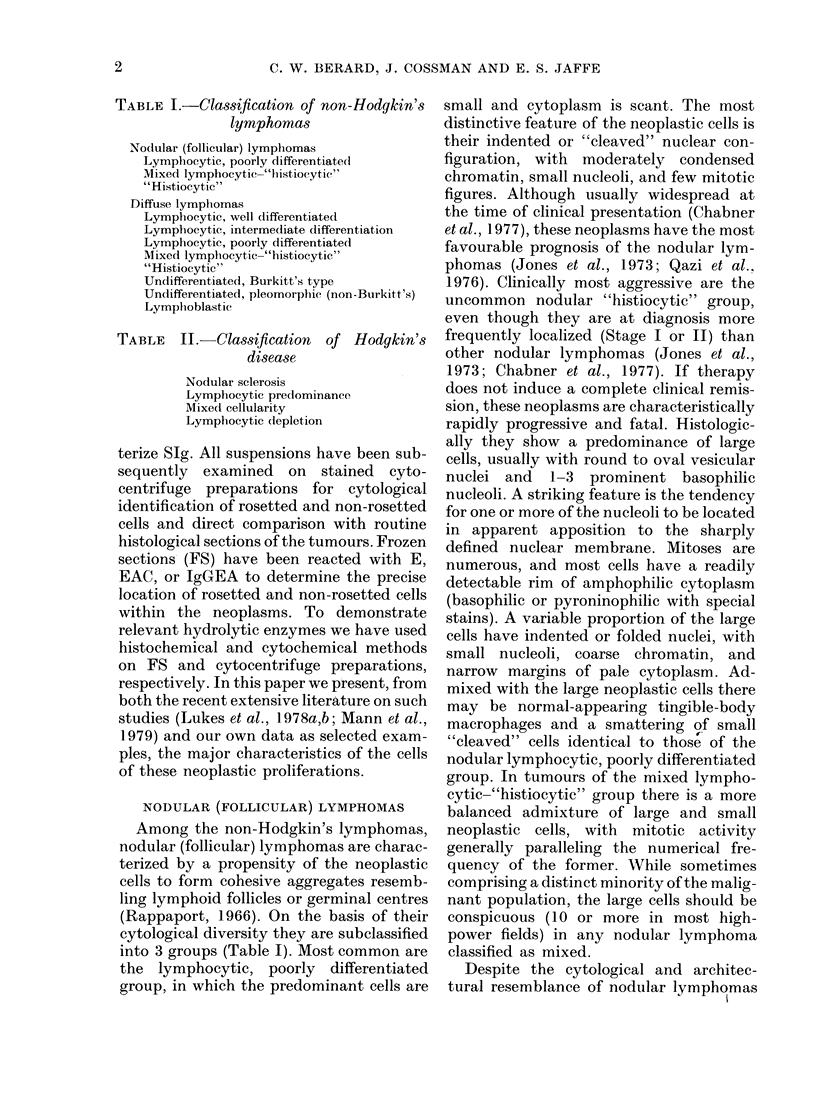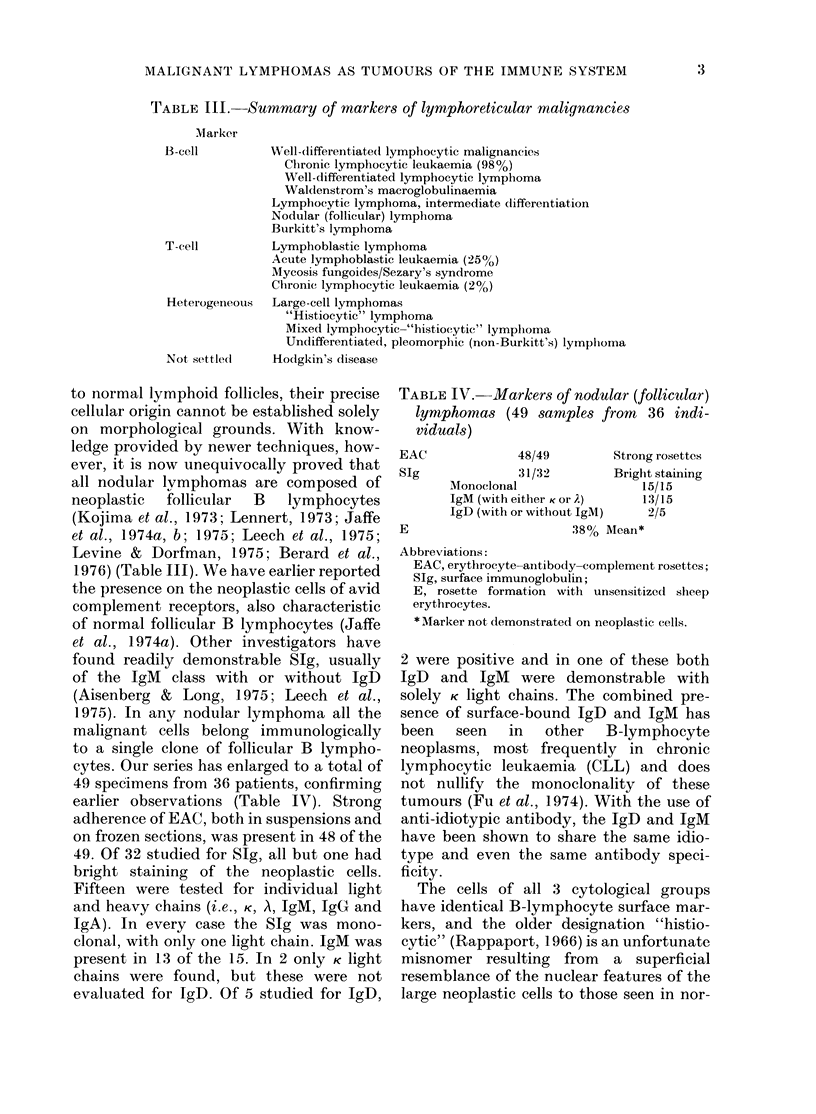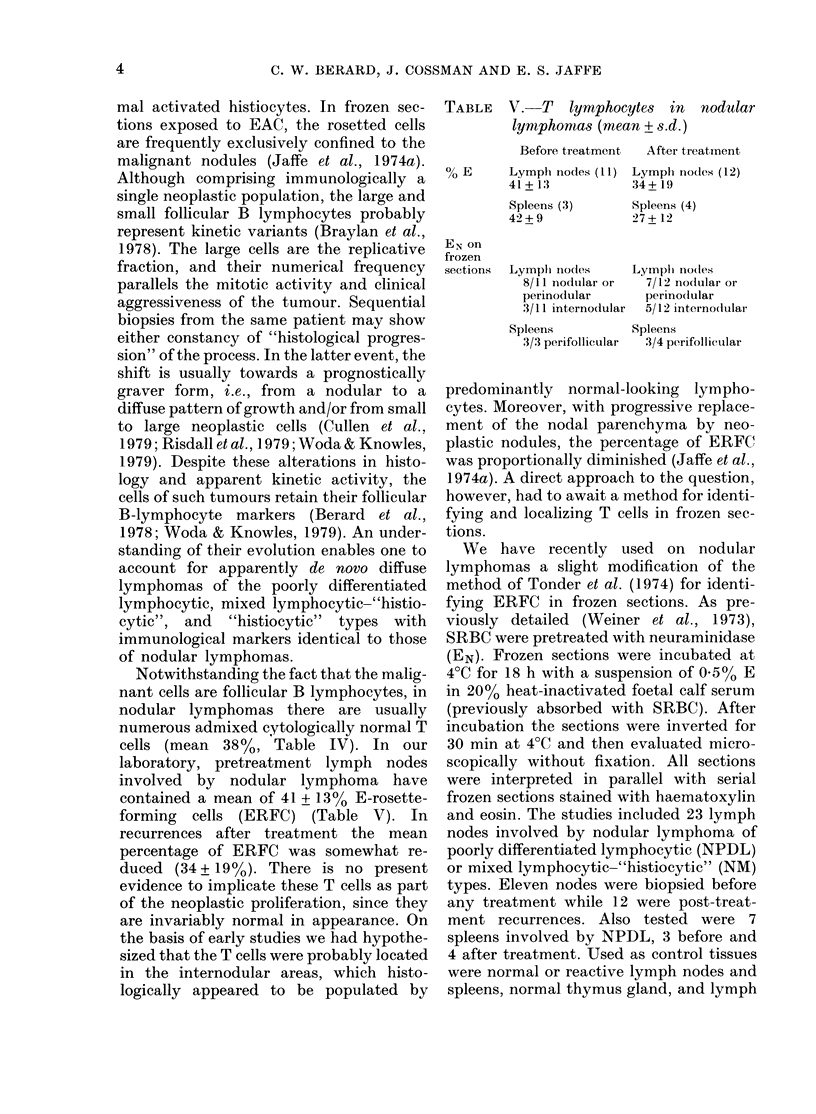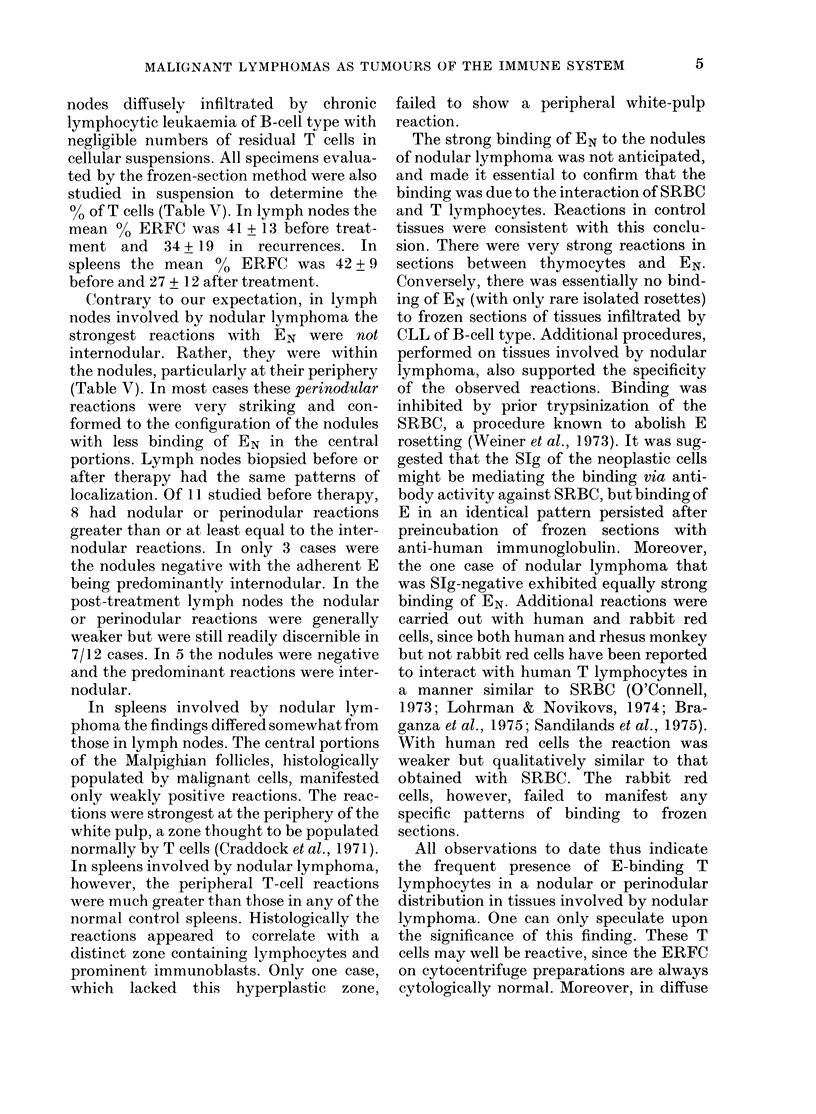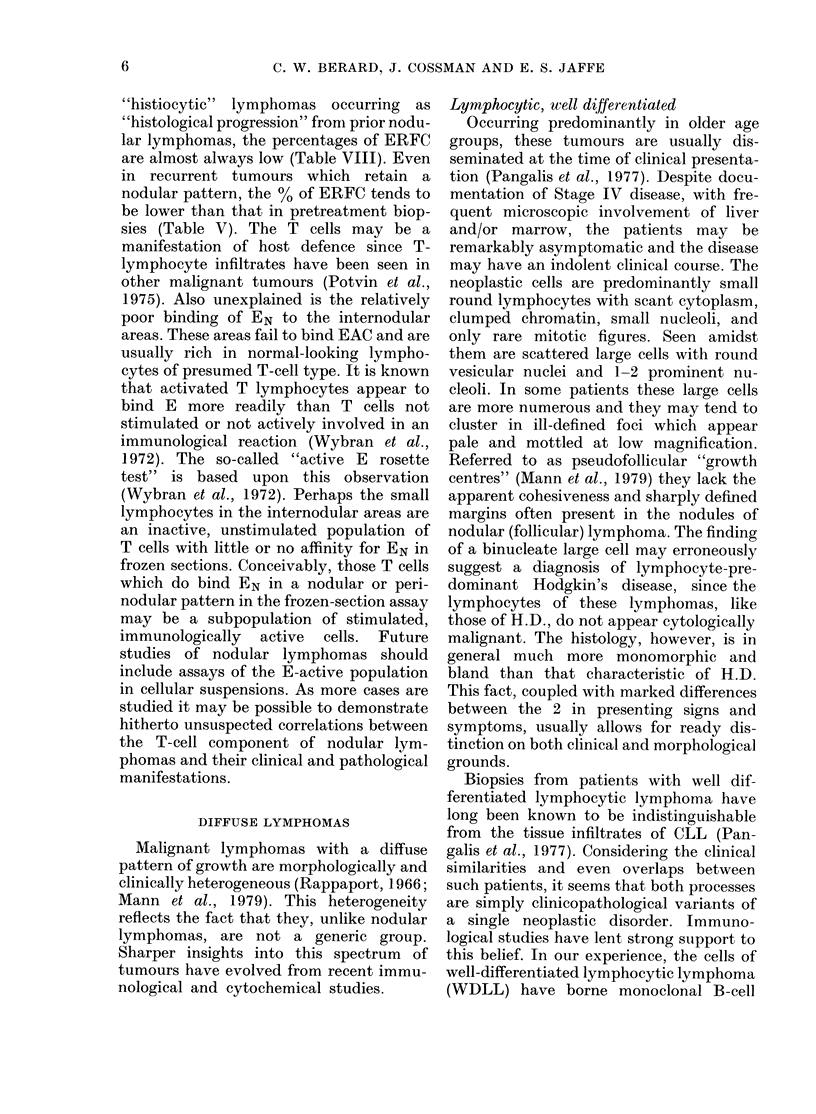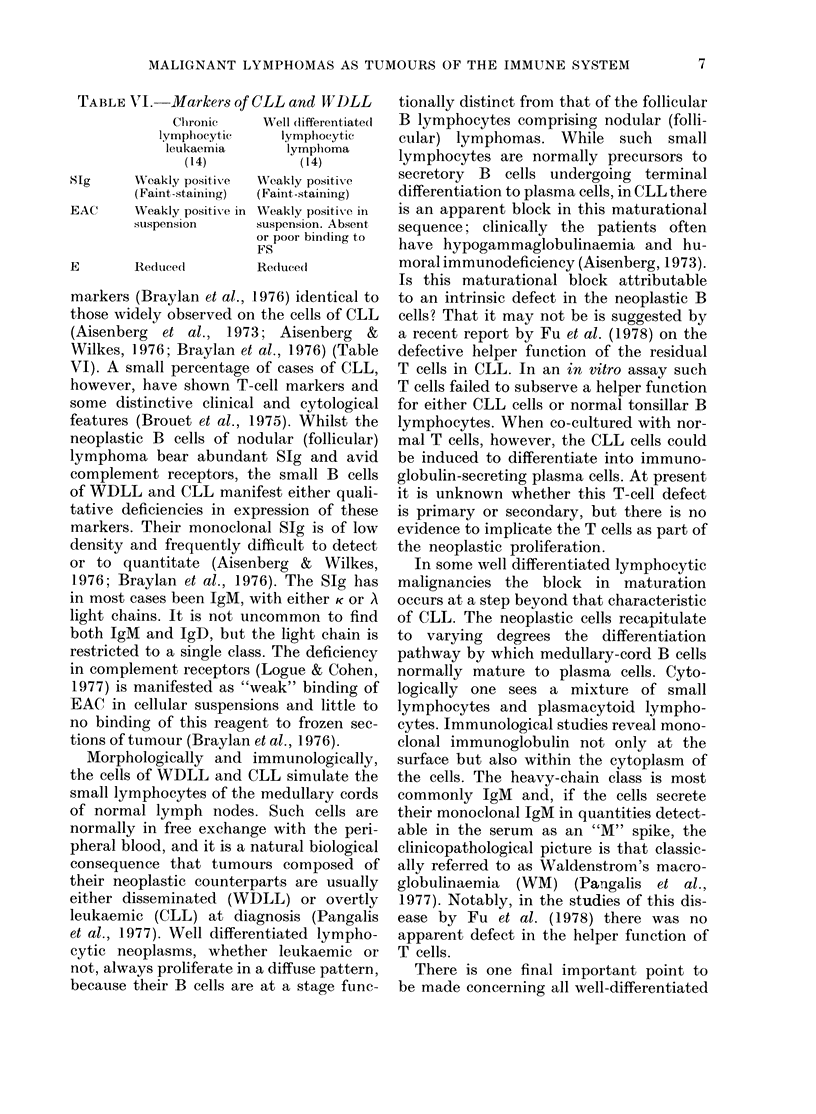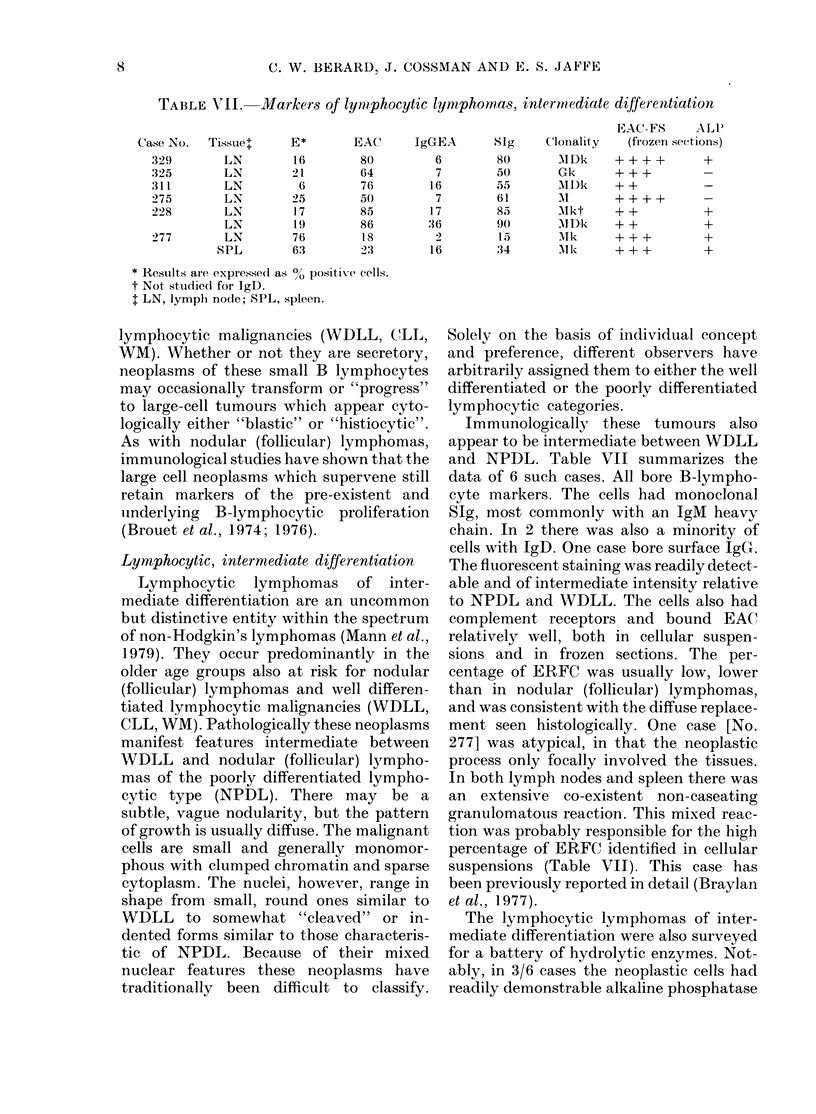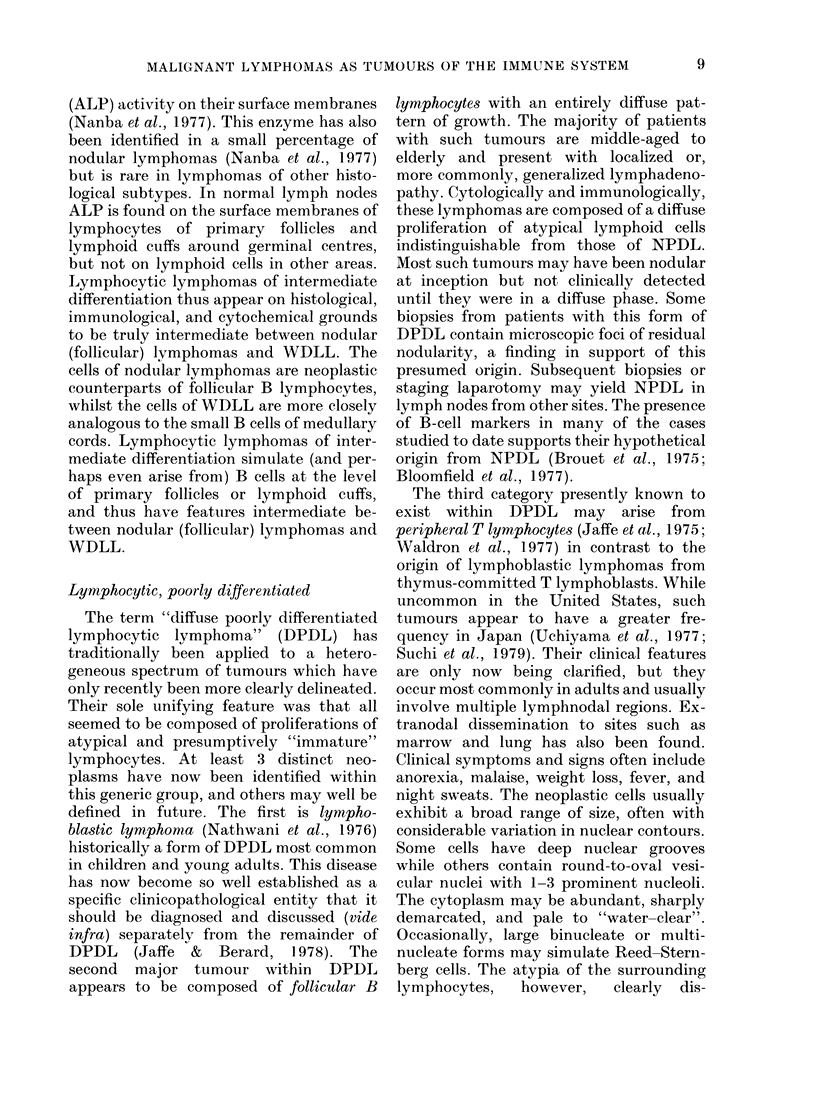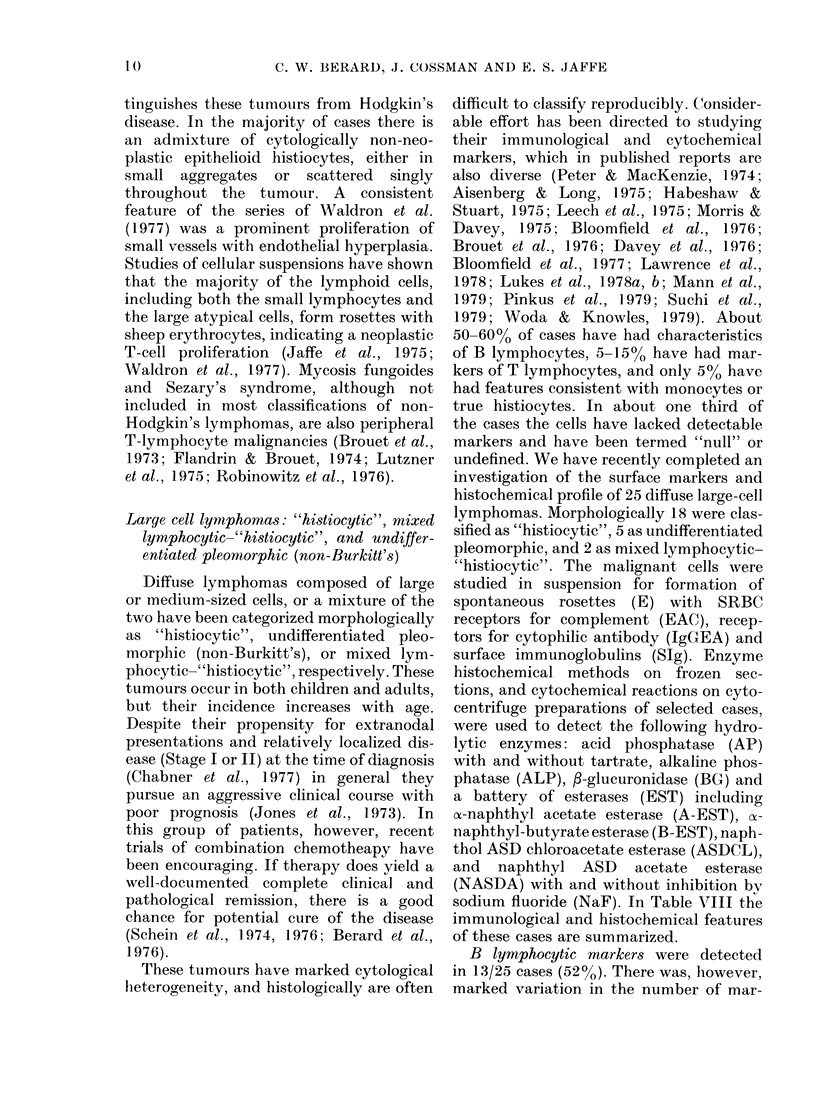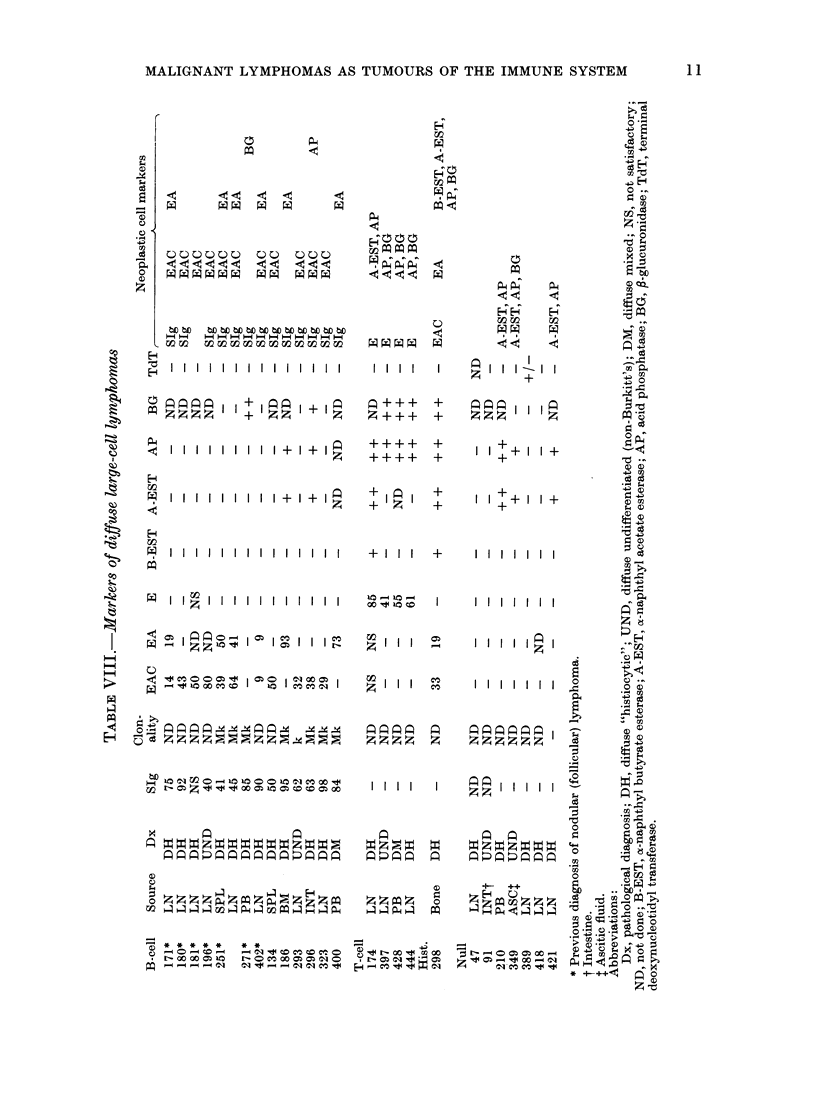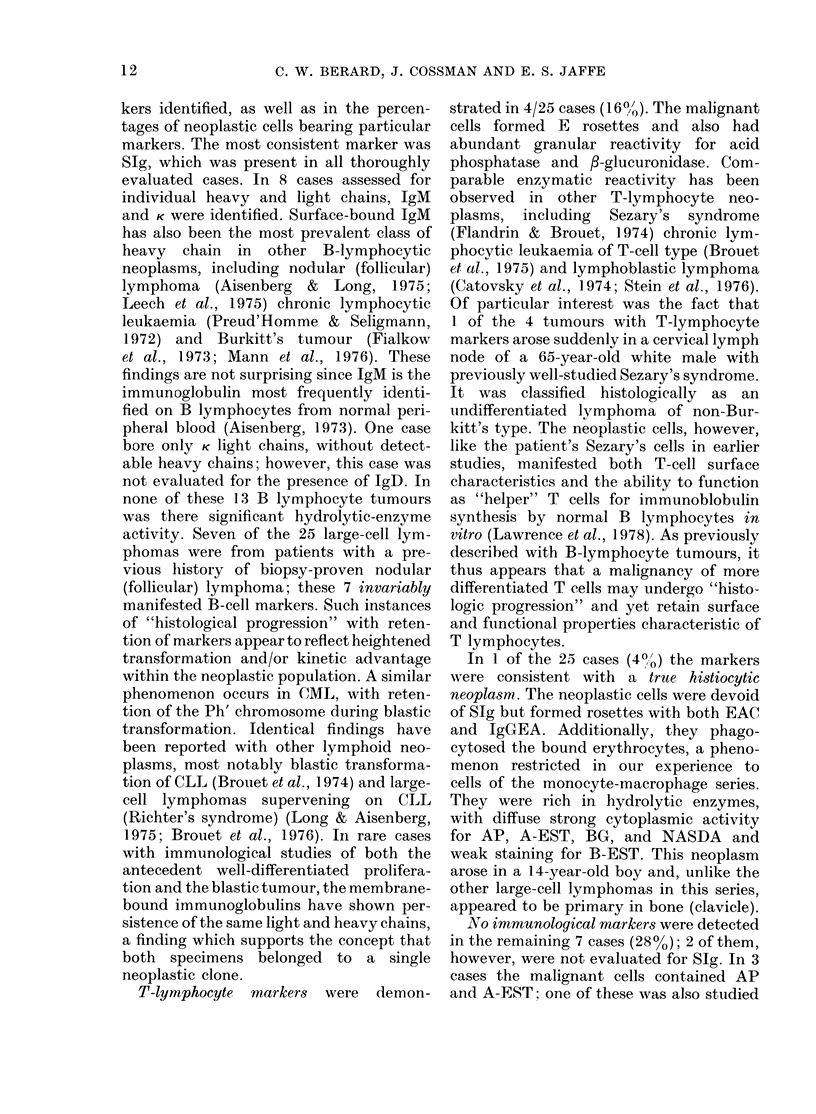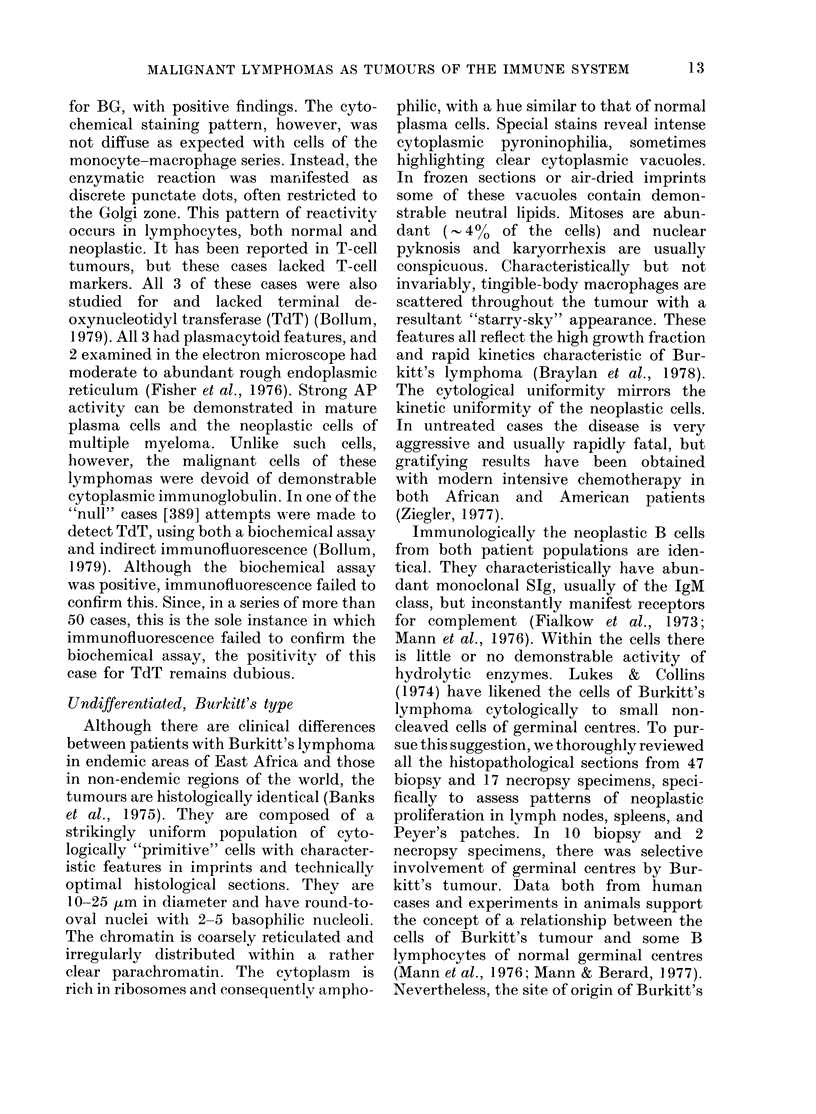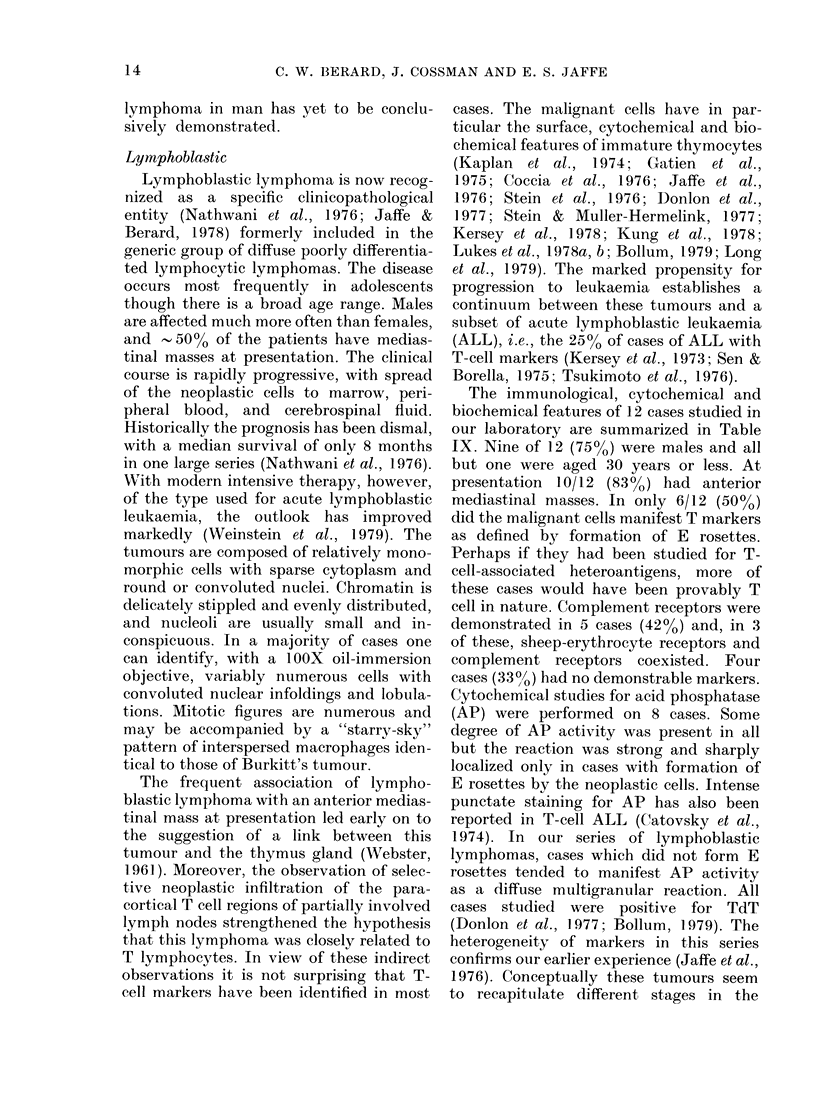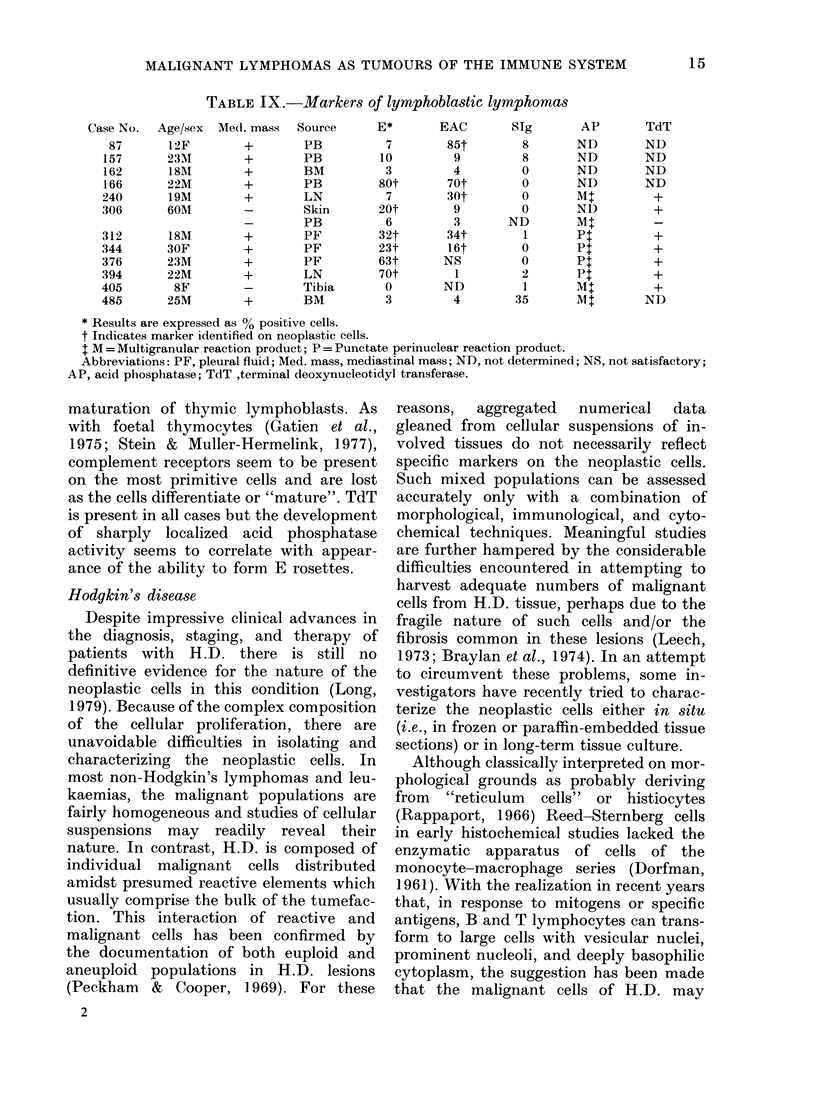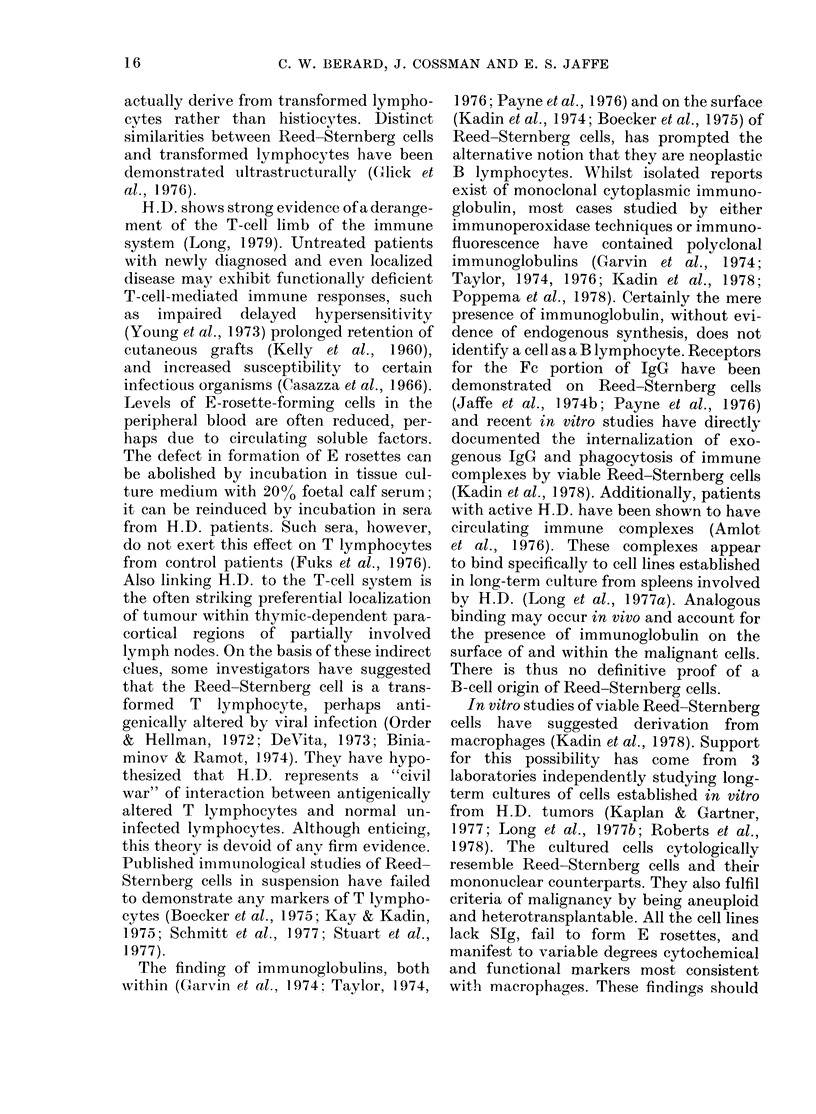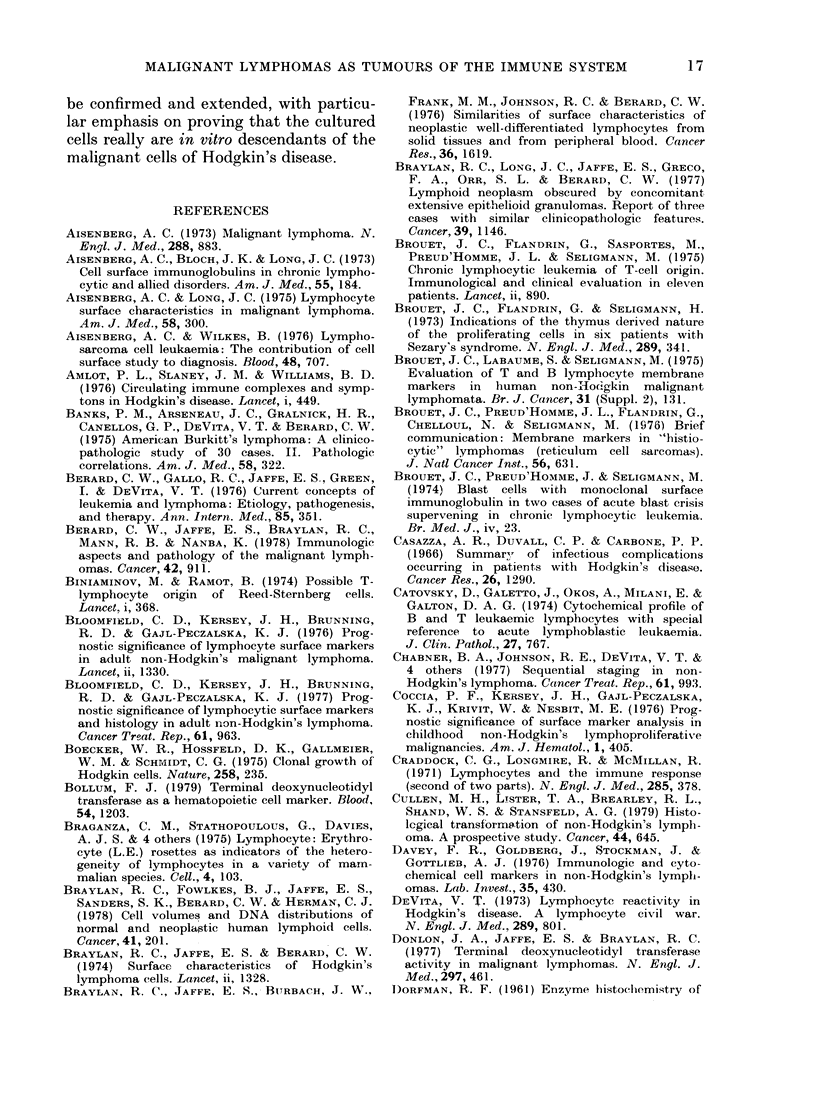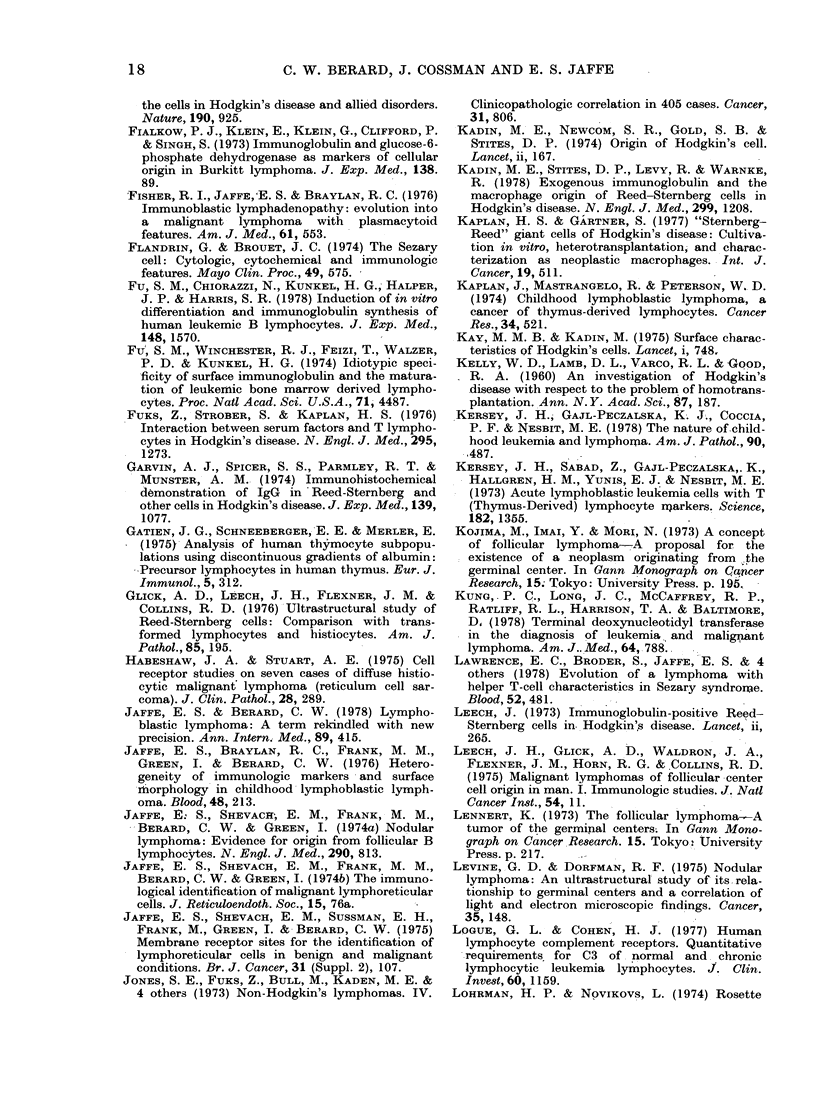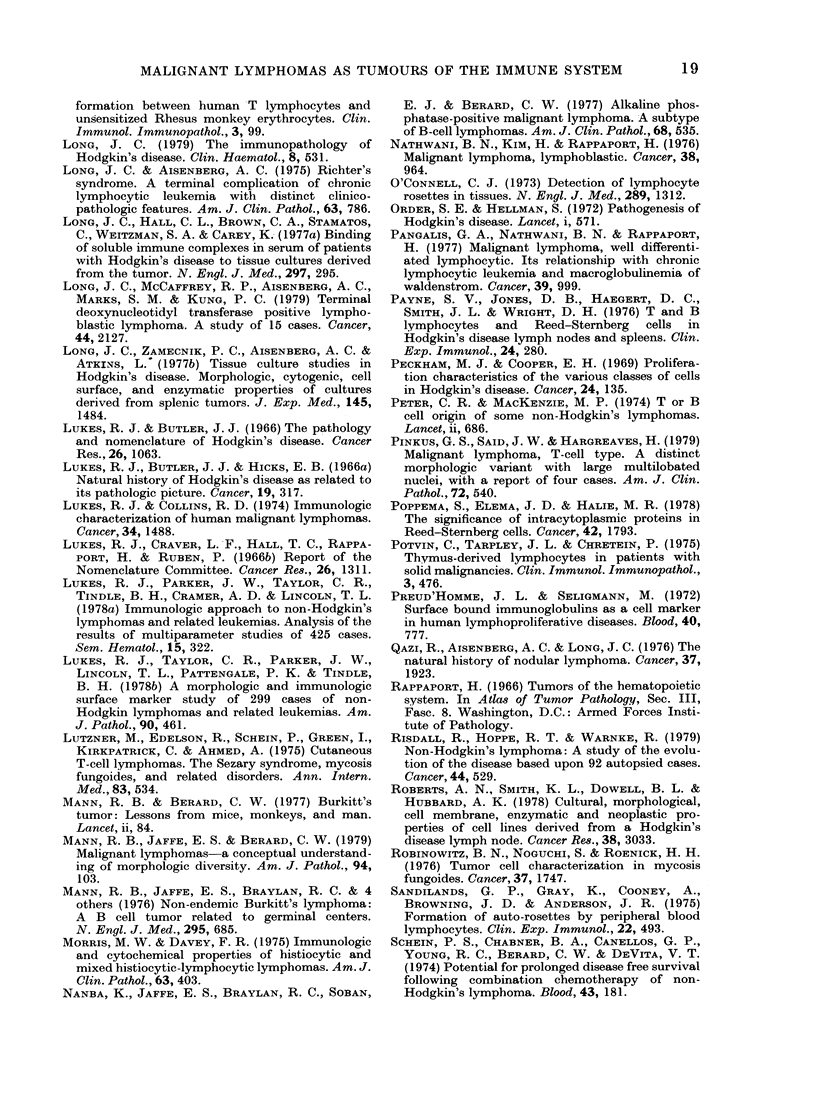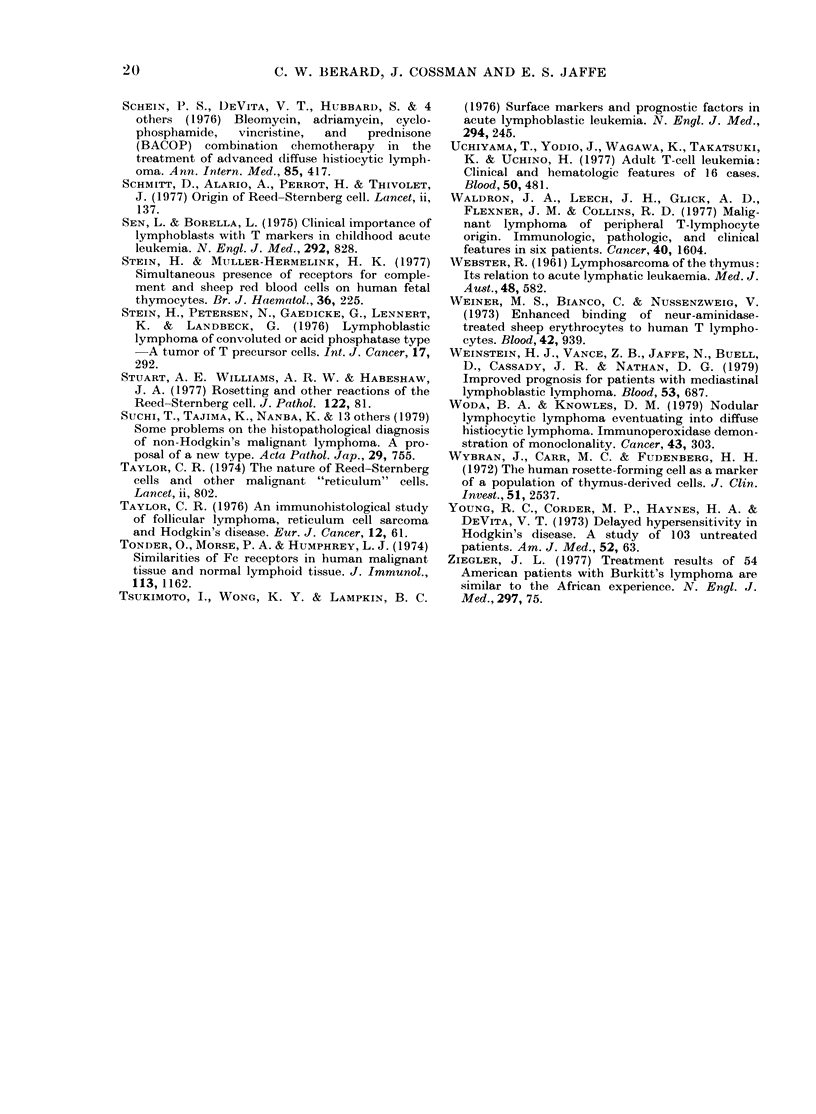# Malignant lymphomas as tumours of the immune system.

**DOI:** 10.1038/bjc.1980.197

**Published:** 1980-07

**Authors:** C. W. Berard, J. Cossman, E. S. Jaffe

## Abstract

Malignant lymphomas have traditionally been classified on solely morphological grounds. With new immunological and cytochemical techniques, it has been possible to characterize normal cells of the T-lymphocyte, B-lymphocyte, and monocyte-macrophage system. Application of these methodologies to malignant lymphomas has established their nature as neoplasms of the immune system. Within the B-lymphocyte system it is possible to identify subpopulations responsible for Burkitt's tumour, follicular (nodular) lymphomas, lymphocytic lymphomas of intermediate differentiation and well differentiated lymphocytic lymphomas. The T-lymphocyte system includes lymphoblastic lymphomas, mycosis fungoides, and Sezary's syndrome. Large-cell lymphomas are diverse, but the majority are tumours of transformed lymphocytes, usually of the B-lymphocyte system. The precise nature of the neoplastic cells of Hodgkin's disease (i.e., Reed-Sternberg cells and their mononuclear counterparts) has not yet been established. Despite previous suggestions of a B-lymphocyte or T-lymphocyte origin, recent studies with in vitro cultivation have strongly suggested derivation from the monocyte-macrophage system.


Br. J. Cancer (1980) 42, 1

Walter Hubert Lecture, delivered 1 April 1980 to the 21st AGM of the BACR by Dr Costan W. Berard

### MALIGNANT LYMPHOMAS AS TUMOURS OF THE IMMUNE SYSTEM

C. W. BERARD, J. COSSMAN AND E. S. JAFFE

*From the Hematopathology Section, Laboratory of Pathology, National Cancer Institute, Bethesda, Maryland 20205, U.S.A.*

**Summary.—Malignant lymphomas have traditionally been classified on solely morphological grounds. With new immunological and cytochemical techniques, it has been possible to characterize normal cells of the T-lymphocyte, B-lymphocyte, and monocyte-macrophage system. Application of these methodologies to malignant lymphomas has established their nature as neoplasms of the immune system. Within the B-lymphocyte system it is possible to identify subpopulations responsible for Burkitt's tumour, follicular (nodular) lymphomas, lymphocytic lymphomas of intermediate differentiation and well differentiated lymphocytic lymphomas. The T-lymphocyte system includes lymphoblastic lymphomas, mycosis fungoides, and Sezary's syndrome. Large-cell lymphomas are diverse, but the majority are tumours of transformed lymphocytes, usually of the B-lymphocyte system. The precise nature of the neoplastic cells of Hodgkin's disease (i.e., Reed–Sternberg cells and their mono-nuclear counterparts) has not yet been established. Despite previous suggestions of a B-lymphocyte or T-lymphocyte origin, recent studies with *in vitro* cultivation have strongly suggested derivation from the monocyte-macrophage system.**

MALIGNANT LYMPHOMAS have been traditionally diagnosed and classified on the basis of solely morphological criteria. Clinicopathological studies have repeatedly shown the applicability and value of the Rappaport classification (Rappaport, 1966) with recent modifications (Nathwani *et al.*, 1976; Pangalis *et al.*, 1977; Mann *et al.*, 1979) (Table I), for the non-Hodgkin's lymphomas. The classification of Hodgkin's disease (HD) originally proffered by Lukes & Butler (1966) and Lukes *et al.* (1966*a*) and later simplified by a nomenclature committee (Lukes *et al.*, 1966*b*) (Table II) has simultaneously gained world-wide acceptance and usage. In recent years, however, new immunological, cytochemical, and ultrastructural techniques have made possible a sophisticated characterization of normal cells of the T-lymphocyte, B-lymphocyte, and monocyte–macrophage series (Mann *et al.*, 1979). Such studies have greatly enhanced current understanding of the compartmentalization, immunology and function of the lymphoreticular system. Use of the same modern methodologies in studies of the neoplastic cells of malignant lymphomas has established that such cells often bear markers similar or identical to those demonstrable on normal cells. Malignant lymphomas are tumours of the immune system (Table III) and they often seem, both structurally and functionally, to be neoplastic caricatures of their normal benign counterparts (Lukes & Collins, 1974; Berard *et al.*, 1976, 1978; Lukes *et al.*, 1978*a*, *b*; Mann *et al.*, 1979).

In our laboratory we have evaluated the neoplastic cells in suspension for spontaneous rosette (E) formation with sheep red blood cells (SRBC), receptors for complement (EAC), receptors for cytophilic antibody (IgGEA), and surface immunoglobulins (SIg). Both polyvalent and monovalent antisera have been used to charac-

TABLE I.—*Classification of non-Hodgkin's lymphomas*

Nodular (follicular) lymphomas
- Lymphocytic, poorly differentiated
- Mixed lymphocytic–"histiocytic"
- "Histiocytic"

Diffuse lymphomas
- Lymphocytic, well differentiated
- Lymphocytic, intermediate differentiation
- Lymphocytic, poorly differentiated
- Mixed lymphocytic–"histiocytic"
- "Histiocytic"
- Undifferentiated, Burkitt's type
- Undifferentiated, pleomorphic (non-Burkitt's)
- Lymphoblastic

TABLE II.—*Classification of Hodgkin's disease*

- Nodular sclerosis

- Lymphocytic predominance

- Mixed cellularity

- Lymphocytic depletion terize SIg. All suspensions have been subsequently examined on stained cytocentrifuge preparations for cytological identification of rosetted and non-rosetted cells and direct comparison with routine histological sections of the tumours. Frozen sections (FS) have been reacted with E, EAC, or IgGEA to determine the precise location of rosetted and non-rosetted cells within the neoplasms. To demonstrate relevant hydrolytic enzymes we have used histochemical and cytochemical methods on FS and cytocentrifuge preparations, respectively. In this paper we present, from both the recent extensive literature on such studies (Lukes *et al.*, 1978*a,b*; Mann *et al.*, 1979) and our own data as selected examples, the major characteristics of the cells of these neoplastic proliferations.

### NODULAR (FOLLICULAR) LYMPHOMAS

Among the non-Hodgkin's lymphomas, nodular (follicular) lymphomas are characterized by a propensity of the neoplastic cells to form cohesive aggregates resembling lymphoid follicles or germinal centres (Rappaport, 1966). On the basis of their cytological diversity they are subclassified into 3 groups (Table I). Most common are the lymphocytic, poorly differentiated group, in which the predominant cells are small and cytoplasm is scant. The most distinctive feature of the neoplastic cells is their indented or "cleaved" nuclear configuration, with moderately condensed chromatin, small nucleoli, and few mitotic figures. Although usually widespread at the time of clinical presentation (Chabner *et al.*, 1977), these neoplasms have the most favourable prognosis of the nodular lymphomas (Jones *et al.*, 1973; Qazi *et al.*, 1976). Clinically most aggressive are the uncommon nodular "histiocytic" group, even though they are at diagnosis more frequently localized (Stage I or II) than other nodular lymphomas (Jones *et al.*, 1973; Chabner *et al.*, 1977). If therapy does not induce a complete clinical remission, these neoplasms are characteristically rapidly progressive and fatal. Histologically they show a predominance of large cells, usually with round to oval vesicular nuclei and 1–3 prominent basophilic nucleoli. A striking feature is the tendency for one or more of the nucleoli to be located in apparent apposition to the sharply defined nuclear membrane. Mitoses are numerous, and most cells have a readily detectable rim of amphophilic cytoplasm (basophilic or pyroninophilic with special stains). A variable proportion of the large cells have indented or folded nuclei, with small nucleoli, coarse chromatin, and narrow margins of pale cytoplasm. Admixed with the large neoplastic cells there may be normal-appearing tingible-body macrophages and a smattering of small "cleaved" cells identical to those of the nodular lymphocytic, poorly differentiated group. In tumours of the mixed lymphocytic–"histiocytic" group there is a more balanced admixture of large and small neoplastic cells, with mitotic activity generally paralleling the numerical frequency of the former. While sometimes comprising a distinct minority of the malignant population, the large cells should be conspicuous (10 or more in most high-power fields) in any nodular lymphoma classified as mixed.

Despite the cytological and architectural resemblance of nodular lymphomas

TABLE III.—*Summary of markers of lymphoreticular malignancies*

| Marker | |
|---|---|
| B-cell | Well-differentiated lymphocytic malignancies<br>  Chronic lymphocytic leukaemia (98%)<br>  Well-differentiated lymphocytic lymphoma<br>  Waldenstrom's macroglobulinaemia<br>Lymphocytic lymphoma, intermediate differentiation<br>Nodular (follicular) lymphoma<br>Burkitt's lymphoma |
| T-cell | Lymphoblastic lymphoma<br>Acute lymphoblastic leukaemia (25%)<br>Mycosis fungoides/Sezary's syndrome<br>Chronic lymphocytic leukaemia (2%) |
| Heterogeneous | Large-cell lymphomas<br>  "Histiocytic" lymphoma<br>  Mixed lymphocytic–"histiocytic" lymphoma<br>  Undifferentiated, pleomorphic (non-Burkitt's) lymphoma |
| Not settled | Hodgkin's disease |

to normal lymphoid follicles, their precise cellular origin cannot be established solely on morphological grounds. With knowledge provided by newer techniques, however, it is now unequivocally proved that all nodular lymphomas are composed of neoplastic follicular B lymphocytes (Kojima *et al.*, 1973; Lennert, 1973; Jaffe *et al.*, 1974*a*, *b*; 1975; Leech *et al.*, 1975; Levine & Dorfman, 1975; Berard *et al.*, 1976) (Table III). We have earlier reported the presence on the neoplastic cells of avid complement receptors, also characteristic of normal follicular B lymphocytes (Jaffe *et al.*, 1974*a*). Other investigators have found readily demonstrable SIg, usually of the IgM class with or without IgD (Aisenberg & Long, 1975; Leech *et al.*, 1975). In any nodular lymphoma all the malignant cells belong immunologically to a single clone of follicular B lymphocytes. Our series has enlarged to a total of 49 specimens from 36 patients, confirming earlier observations (Table IV). Strong adherence of EAC, both in suspensions and on frozen sections, was present in 48 of the 49. Of 32 studied for SIg, all but one had bright staining of the neoplastic cells. Fifteen were tested for individual light and heavy chains (*i.e.*, $\kappa$, $\lambda$, IgM, IgG and IgA). In every case the SIg was monoclonal, with only one light chain. IgM was present in 13 of the 15. In 2 only $\kappa$ light chains were found, but these were not evaluated for IgD. Of 5 studied for IgD,

TABLE IV.—*Markers of nodular (follicular) lymphomas (49 samples from 36 individuals)*

| | | | |
|---|---|---|---|
| EAC | 48/49 | | Strong rosettes |
| SIg | 31/32 | | Bright staining |
| | Monoclonal | | 15/15 |
| | IgM (with either $\kappa$ or $\lambda$) | | 13/15 |
| | IgD (with or without IgM) | | 2/5 |
| E | | 38% | Mean* |

Abbreviations:
EAC, erythrocyte–antibody–complement rosettes;
SIg, surface immunoglobulin;
E, rosette formation with unsensitized sheep erythrocytes.

*Marker not demonstrated on neoplastic cells.

2 were positive and in one of these both IgD and IgM were demonstrable with solely $\kappa$ light chains. The combined presence of surface-bound IgD and IgM has been seen in other B-lymphocyte neoplasms, most frequently in chronic lymphocytic leukaemia (CLL) and does not nullify the monoclonality of these tumours (Fu *et al.*, 1974). With the use of anti-idiotypic antibody, the IgD and IgM have been shown to share the same idiotype and even the same antibody specificity.

The cells of all 3 cytological groups have identical B-lymphocyte surface markers, and the older designation "histiocytic" (Rappaport, 1966) is an unfortunate misnomer resulting from a superficial resemblance of the nuclear features of the large neoplastic cells to those seen in normal activated histiocytes. In frozen sections exposed to EAC, the rosetted cells are frequently exclusively confined to the malignant nodules (Jaffe *et al.*, 1974*a*). Although comprising immunologically a single neoplastic population, the large and small follicular B lymphocytes probably represent kinetic variants (Braylan *et al.*, 1978). The large cells are the replicative fraction, and their numerical frequency parallels the mitotic activity and clinical aggressiveness of the tumour. Sequential biopsies from the same patient may show either constancy of "histological progression" of the process. In the latter event, the shift is usually towards a prognostically graver form, *i.e.*, from a nodular to a diffuse pattern of growth and/or from small to large neoplastic cells (Cullen *et al.*, 1979; Risdall *et al.*, 1979; Woda & Knowles, 1979). Despite these alterations in histology and apparent kinetic activity, the cells of such tumours retain their follicular B-lymphocyte markers (Berard *et al.*, 1978; Woda & Knowles, 1979). An understanding of their evolution enables one to account for apparently *de novo* diffuse lymphomas of the poorly differentiated lymphocytic, mixed lymphocytic–"histiocytic", and "histiocytic" types with immunological markers identical to those of nodular lymphomas.

Notwithstanding the fact that the malignant cells are follicular B lymphocytes, in nodular lymphomas there are usually numerous admixed cytologically normal T cells (mean 38%, Table IV). In our laboratory, pretreatment lymph nodes involved by nodular lymphoma have contained a mean of 41 ± 13% E-rosette-forming cells (ERFC) (Table V). In recurrences after treatment the mean percentage of ERFC was somewhat reduced (34 ± 19%). There is no present evidence to implicate these T cells as part of the neoplastic proliferation, since they are invariably normal in appearance. On the basis of early studies we had hypothesized that the T cells were probably located in the internodular areas, which histologically appeared to be populated by

TABLE V.—*T lymphocytes in nodular lymphomas* (*mean* ± *s.d.*)

| | Before treatment | After treatment |
|---|---|---|
| % E | Lymph nodes (11)<br>41 ± 13 | Lymph nodes (12)<br>34 ± 19 |
| | Spleens (3)<br>42 ± 9 | Spleens (4)<br>27 ± 12 |
| $E_N$ on frozen sections | Lymph nodes<br>8/11 nodular or perinodular<br>3/11 internodular | Lymph nodes<br>7/12 nodular or perinodular<br>5/12 internodular |
| | Spleens<br>3/3 perifollicular | Spleens<br>3/4 perifollicular |

predominantly normal-looking lymphocytes. Moreover, with progressive replacement of the nodal parenchyma by neoplastic nodules, the percentage of ERFC was proportionally diminished (Jaffe *et al.*, 1974*a*). A direct approach to the question, however, had to await a method for identifying and localizing T cells in frozen sections.

We have recently used on nodular lymphomas a slight modification of the method of Tonder *et al.* (1974) for identifying ERFC in frozen sections. As previously detailed (Weiner *et al.*, 1973), SRBC were pretreated with neuraminidase ($E_N$). Frozen sections were incubated at 4°C for 18 h with a suspension of 0·5% E in 20% heat-inactivated foetal calf serum (previously absorbed with SRBC). After incubation the sections were inverted for 30 min at 4°C and then evaluated microscopically without fixation. All sections were interpreted in parallel with serial frozen sections stained with haematoxylin and eosin. The studies included 23 lymph nodes involved by nodular lymphoma of poorly differentiated lymphocytic (NPDL) or mixed lymphocytic–"histiocytic" (NM) types. Eleven nodes were biopsied before any treatment while 12 were post-treatment recurrences. Also tested were 7 spleens involved by NPDL, 3 before and 4 after treatment. Used as control tissues were normal or reactive lymph nodes and spleens, normal thymus gland, and lymph nodes diffusely infiltrated by chronic lymphocytic leukaemia of B-cell type with negligible numbers of residual T cells in cellular suspensions. All specimens evaluated by the frozen-section method were also studied in suspension to determine the % of T cells (Table V). In lymph nodes the mean % ERFC was $41 \pm 13$ before treatment and $34 \pm 19$ in recurrences. In spleens the mean % ERFC was $42 \pm 9$ before and $27 \pm 12$ after treatment.

Contrary to our expectation, in lymph nodes involved by nodular lymphoma the strongest reactions with $E_N$ were *not* internodular. Rather, they were within the nodules, particularly at their periphery (Table V). In most cases these *perinodular* reactions were very striking and conformed to the configuration of the nodules with less binding of $E_N$ in the central portions. Lymph nodes biopsied before or after therapy had the same patterns of localization. Of 11 studied before therapy, 8 had nodular or perinodular reactions greater than or at least equal to the internodular reactions. In only 3 cases were the nodules negative with the adherent E being predominantly internodular. In the post-treatment lymph nodes the nodular or perinodular reactions were generally weaker but were still readily discernible in 7/12 cases. In 5 the nodules were negative and the predominant reactions were internodular.

In spleens involved by nodular lymphoma the findings differed somewhat from those in lymph nodes. The central portions of the Malpighian follicles, histologically populated by malignant cells, manifested only weakly positive reactions. The reactions were strongest at the periphery of the white pulp, a zone thought to be populated normally by T cells (Craddock *et al.*, 1971). In spleens involved by nodular lymphoma, however, the peripheral T-cell reactions were much greater than those in any of the normal control spleens. Histologically the reactions appeared to correlate with a distinct zone containing lymphocytes and prominent immunoblasts. Only one case, which lacked this hyperplastic zone, failed to show a peripheral white-pulp reaction.

The strong binding of $E_N$ to the nodules of nodular lymphoma was not anticipated, and made it essential to confirm that the binding was due to the interaction of SRBC and T lymphocytes. Reactions in control tissues were consistent with this conclusion. There were very strong reactions in sections between thymocytes and $E_N$. Conversely, there was essentially no binding of $E_N$ (with only rare isolated rosettes) to frozen sections of tissues infiltrated by CLL of B-cell type. Additional procedures, performed on tissues involved by nodular lymphoma, also supported the specificity of the observed reactions. Binding was inhibited by prior trypsinization of the SRBC, a procedure known to abolish E rosetting (Weiner *et al.*, 1973). It was suggested that the SIg of the neoplastic cells might be mediating the binding *via* antibody activity against SRBC, but binding of E in an identical pattern persisted after preincubation of frozen sections with anti-human immunoglobulin. Moreover, the one case of nodular lymphoma that was SIg-negative exhibited equally strong binding of $E_N$. Additional reactions were carried out with human and rabbit red cells, since both human and rhesus monkey but not rabbit red cells have been reported to interact with human T lymphocytes in a manner similar to SRBC (O'Connell, 1973; Lohrman & Novikovs, 1974; Braganza *et al.*, 1975; Sandilands *et al.*, 1975). With human red cells the reaction was weaker but qualitatively similar to that obtained with SRBC. The rabbit red cells, however, failed to manifest any specific patterns of binding to frozen sections.

All observations to date thus indicate the frequent presence of E-binding T lymphocytes in a nodular or perinodular distribution in tissues involved by nodular lymphoma. One can only speculate upon the significance of this finding. These T cells may well be reactive, since the ERFC on cytocentrifuge preparations are always cytologically normal. Moreover, in diffuse

"histiocytic" lymphomas occurring as "histological progression" from prior nodular lymphomas, the percentages of ERFC are almost always low (Table VIII). Even in recurrent tumours which retain a nodular pattern, the % of ERFC tends to be lower than that in pretreatment biopsies (Table V). The T cells may be a manifestation of host defence since T-lymphocyte infiltrates have been seen in other malignant tumours (Potvin *et al.*, 1975). Also unexplained is the relatively poor binding of $E_N$ to the internodular areas. These areas fail to bind EAC and are usually rich in normal-looking lymphocytes of presumed T-cell type. It is known that activated T lymphocytes appear to bind E more readily than T cells not stimulated or not actively involved in an immunological reaction (Wybran *et al.*, 1972). The so-called "active E rosette test" is based upon this observation (Wybran *et al.*, 1972). Perhaps the small lymphocytes in the internodular areas are an inactive, unstimulated population of T cells with little or no affinity for $E_N$ in frozen sections. Conceivably, those T cells which do bind $E_N$ in a nodular or perinodular pattern in the frozen-section assay may be a subpopulation of stimulated, immunologically active cells. Future studies of nodular lymphomas should include assays of the E-active population in cellular suspensions. As more cases are studied it may be possible to demonstrate hitherto unsuspected correlations between the T-cell component of nodular lymphomas and their clinical and pathological manifestations.

### DIFFUSE LYMPHOMAS

Malignant lymphomas with a diffuse pattern of growth are morphologically and clinically heterogeneous (Rappaport, 1966; Mann *et al.*, 1979). This heterogeneity reflects the fact that they, unlike nodular lymphomas, are not a generic group. Sharper insights into this spectrum of tumours have evolved from recent immunological and cytochemical studies.

### *Lymphocytic, well differentiated*

Occurring predominantly in older age groups, these tumours are usually disseminated at the time of clinical presentation (Pangalis *et al.*, 1977). Despite documentation of Stage IV disease, with frequent microscopic involvement of liver and/or marrow, the patients may be remarkably asymptomatic and the disease may have an indolent clinical course. The neoplastic cells are predominantly small round lymphocytes with scant cytoplasm, clumped chromatin, small nucleoli, and only rare mitotic figures. Seen amidst them are scattered large cells with round vesicular nuclei and 1–2 prominent nucleoli. In some patients these large cells are more numerous and they may tend to cluster in ill-defined foci which appear pale and mottled at low magnification. Referred to as pseudofollicular "growth centres" (Mann *et al.*, 1979) they lack the apparent cohesiveness and sharply defined margins often present in the nodules of nodular (follicular) lymphoma. The finding of a binucleate large cell may erroneously suggest a diagnosis of lymphocyte-predominant Hodgkin's disease, since the lymphocytes of these lymphomas, like those of H.D., do not appear cytologically malignant. The histology, however, is in general much more monomorphic and bland than that characteristic of H.D. This fact, coupled with marked differences between the 2 in presenting signs and symptoms, usually allows for ready distinction on both clinical and morphological grounds.

Biopsies from patients with well differentiated lymphocytic lymphoma have long been known to be indistinguishable from the tissue infiltrates of CLL (Pangalis *et al.*, 1977). Considering the clinical similarities and even overlaps between such patients, it seems that both processes are simply clinicopathological variants of a single neoplastic disorder. Immunological studies have lent strong support to this belief. In our experience, the cells of well-differentiated lymphocytic lymphoma (WDLL) have borne monoclonal B-cell

TABLE VI.—*Markers of CLL and WDLL*

| | Chronic lymphocytic leukaemia (14) | Well differentiated lymphocytic lymphoma (14) |
|---|---|---|
| SIg | Weakly positive (Faint-staining) | Weakly positive (Faint-staining) |
| EAC | Weakly positive in suspension | Weakly positive in suspension. Absent or poor binding to FS |
| E | Reduced | Reduced |

markers (Braylan *et al.*, 1976) identical to those widely observed on the cells of CLL (Aisenberg *et al.*, 1973; Aisenberg & Wilkes, 1976; Braylan *et al.*, 1976) (Table VI). A small percentage of cases of CLL, however, have shown T-cell markers and some distinctive clinical and cytological features (Brouet *et al.*, 1975). Whilst the neoplastic B cells of nodular (follicular) lymphoma bear abundant SIg and avid complement receptors, the small B cells of WDLL and CLL manifest either qualitative deficiencies in expression of these markers. Their monoclonal SIg is of low density and frequently difficult to detect or to quantitate (Aisenberg & Wilkes, 1976; Braylan *et al.*, 1976). The SIg has in most cases been IgM, with either $\kappa$ or $\lambda$ light chains. It is not uncommon to find both IgM and IgD, but the light chain is restricted to a single class. The deficiency in complement receptors (Logue & Cohen, 1977) is manifested as "weak" binding of EAC in cellular suspensions and little to no binding of this reagent to frozen sections of tumour (Braylan *et al.*, 1976).

Morphologically and immunologically, the cells of WDLL and CLL simulate the small lymphocytes of the medullary cords of normal lymph nodes. Such cells are normally in free exchange with the peripheral blood, and it is a natural biological consequence that tumours composed of their neoplastic counterparts are usually either disseminated (WDLL) or overtly leukaemic (CLL) at diagnosis (Pangalis *et al.*, 1977). Well differentiated lymphocytic neoplasms, whether leukaemic or not, always proliferate in a diffuse pattern, because their B cells are at a stage functionally distinct from that of the follicular B lymphocytes comprising nodular (follicular) lymphomas. While such small lymphocytes are normally precursors to secretory B cells undergoing terminal differentiation to plasma cells, in CLL there is an apparent block in this maturational sequence; clinically the patients often have hypogammaglobulinaemia and humoral immunodeficiency (Aisenberg, 1973). Is this maturational block attributable to an intrinsic defect in the neoplastic B cells? That it may not be is suggested by a recent report by Fu *et al.* (1978) on the defective helper function of the residual T cells in CLL. In an *in vitro* assay such T cells failed to subserve a helper function for either CLL cells or normal tonsillar B lymphocytes. When co-cultured with normal T cells, however, the CLL cells could be induced to differentiate into immunoglobulin-secreting plasma cells. At present it is unknown whether this T-cell defect is primary or secondary, but there is no evidence to implicate the T cells as part of the neoplastic proliferation.

In some well differentiated lymphocytic malignancies the block in maturation occurs at a step beyond that characteristic of CLL. The neoplastic cells recapitulate to varying degrees the differentiation pathway by which medullary-cord B cells normally mature to plasma cells. Cytologically one sees a mixture of small lymphocytes and plasmacytoid lymphocytes. Immunological studies reveal monoclonal immunoglobulin not only at the surface but also within the cytoplasm of the cells. The heavy-chain class is most commonly IgM and, if the cells secrete their monoclonal IgM in quantities detectable in the serum as an "M" spike, the clinicopathological picture is that classically referred to as Waldenstrom's macroglobulinaemia (WM) (Pangalis *et al.*, 1977). Notably, in the studies of this disease by Fu *et al.* (1978) there was no apparent defect in the helper function of T cells.

There is one final important point to be made concerning all well-differentiated

TABLE VII.—*Markers of lymphocytic lymphomas, intermediate differentiation*

| Case No. | Tissue‡ | E* | EAC | IgGEA | SIg | Clonality | EAC-FS (frozen sections) | ALP (frozen sections) |
|---|---|---|---|---|---|---|---|---|
| 329 | LN | 16 | 80 | 6 | 80 | MDk | + + + + | + |
| 325 | LN | 21 | 64 | 7 | 50 | Gk | + + + | − |
| 311 | LN | 6 | 76 | 16 | 55 | MDk | + + | − |
| 275 | LN | 25 | 50 | 7 | 61 | M | + + + + | − |
| 228 | LN | 17 | 85 | 17 | 85 | Mk† | + + | + |
| | LN | 19 | 86 | 36 | 90 | MDk | + + | + |
| 277 | LN | 76 | 18 | 2 | 15 | Mk | + + + | + |
| | SPL | 63 | 23 | 16 | 34 | Mk | + + + | + |

* Results are expressed as % positive cells.
† Not studied for IgD.
‡ LN, lymph node; SPL, spleen.

lymphocytic malignancies (WDLL, CLL, WM). Whether or not they are secretory, neoplasms of these small B lymphocytes may occasionally transform or "progress" to large-cell tumours which appear cytologically either "blastic" or "histiocytic". As with nodular (follicular) lymphomas, immunological studies have shown that the large cell neoplasms which supervene still retain markers of the pre-existent and underlying B-lymphocytic proliferation (Brouet *et al.*, 1974; 1976).

*Lymphocytic, intermediate differentiation*

Lymphocytic lymphomas of intermediate differentiation are an uncommon but distinctive entity within the spectrum of non-Hodgkin's lymphomas (Mann *et al.*, 1979). They occur predominantly in the older age groups also at risk for nodular (follicular) lymphomas and well differentiated lymphocytic malignancies (WDLL, CLL, WM). Pathologically these neoplasms manifest features intermediate between WDLL and nodular (follicular) lymphomas of the poorly differentiated lymphocytic type (NPDL). There may be a subtle, vague nodularity, but the pattern of growth is usually diffuse. The malignant cells are small and generally monomorphous with clumped chromatin and sparse cytoplasm. The nuclei, however, range in shape from small, round ones similar to WDLL to somewhat "cleaved" or indented forms similar to those characteristic of NPDL. Because of their mixed nuclear features these neoplasms have traditionally been difficult to classify. Solely on the basis of individual concept and preference, different observers have arbitrarily assigned them to either the well differentiated or the poorly differentiated lymphocytic categories.

Immunologically these tumours also appear to be intermediate between WDLL and NPDL. Table VII summarizes the data of 6 such cases. All bore B-lymphocyte markers. The cells had monoclonal SIg, most commonly with an IgM heavy chain. In 2 there was also a minority of cells with IgD. One case bore surface IgG. The fluorescent staining was readily detectable and of intermediate intensity relative to NPDL and WDLL. The cells also had complement receptors and bound EAC relatively well, both in cellular suspensions and in frozen sections. The percentage of ERFC was usually low, lower than in nodular (follicular) lymphomas, and was consistent with the diffuse replacement seen histologically. One case [No. 277] was atypical, in that the neoplastic process only focally involved the tissues. In both lymph nodes and spleen there was an extensive co-existent non-caseating granulomatous reaction. This mixed reaction was probably responsible for the high percentage of ERFC identified in cellular suspensions (Table VII). This case has been previously reported in detail (Braylan *et al.*, 1977).

The lymphocytic lymphomas of intermediate differentiation were also surveyed for a battery of hydrolytic enzymes. Notably, in 3/6 cases the neoplastic cells had readily demonstrable alkaline phosphatase

(ALP) activity on their surface membranes (Nanba *et al.*, 1977). This enzyme has also been identified in a small percentage of nodular lymphomas (Nanba *et al.*, 1977) but is rare in lymphomas of other histological subtypes. In normal lymph nodes ALP is found on the surface membranes of lymphocytes of primary follicles and lymphoid cuffs around germinal centres, but not on lymphoid cells in other areas. Lymphocytic lymphomas of intermediate differentiation thus appear on histological, immunological, and cytochemical grounds to be truly intermediate between nodular (follicular) lymphomas and WDLL. The cells of nodular lymphomas are neoplastic counterparts of follicular B lymphocytes, whilst the cells of WDLL are more closely analogous to the small B cells of medullary cords. Lymphocytic lymphomas of intermediate differentiation simulate (and perhaps even arise from) B cells at the level of primary follicles or lymphoid cuffs, and thus have features intermediate between nodular (follicular) lymphomas and WDLL.

*Lymphocytic, poorly differentiated*

The term "diffuse poorly differentiated lymphocytic lymphoma" (DPDL) has traditionally been applied to a heterogeneous spectrum of tumours which have only recently been more clearly delineated. Their sole unifying feature was that all seemed to be composed of proliferations of atypical and presumptively "immature" lymphocytes. At least 3 distinct neoplasms have now been identified within this generic group, and others may well be defined in future. The first is *lymphoblastic lymphoma* (Nathwani *et al.*, 1976) historically a form of DPDL most common in children and young adults. This disease has now become so well established as a specific clinicopathological entity that it should be diagnosed and discussed (*vide infra*) separately from the remainder of DPDL (Jaffe & Berard, 1978). The second major tumour within DPDL appears to be composed of *follicular B lymphocytes* with an entirely diffuse pattern of growth. The majority of patients with such tumours are middle-aged to elderly and present with localized or, more commonly, generalized lymphadenopathy. Cytologically and immunologically, these lymphomas are composed of a diffuse proliferation of atypical lymphoid cells indistinguishable from those of NPDL. Most such tumours may have been nodular at inception but not clinically detected until they were in a diffuse phase. Some biopsies from patients with this form of DPDL contain microscopic foci of residual nodularity, a finding in support of this presumed origin. Subsequent biopsies or staging laparotomy may yield NPDL in lymph nodes from other sites. The presence of B-cell markers in many of the cases studied to date supports their hypothetical origin from NPDL (Brouet *et al.*, 1975; Bloomfield *et al.*, 1977).

The third category presently known to exist within DPDL may arise from *peripheral T lymphocytes* (Jaffe *et al.*, 1975; Waldron *et al.*, 1977) in contrast to the origin of lymphoblastic lymphomas from thymus-committed T lymphoblasts. While uncommon in the United States, such tumours appear to have a greater frequency in Japan (Uchiyama *et al.*, 1977; Suchi *et al.*, 1979). Their clinical features are only now being clarified, but they occur most commonly in adults and usually involve multiple lymphnodal regions. Extranodal dissemination to sites such as marrow and lung has also been found. Clinical symptoms and signs often include anorexia, malaise, weight loss, fever, and night sweats. The neoplastic cells usually exhibit a broad range of size, often with considerable variation in nuclear contours. Some cells have deep nuclear grooves while others contain round-to-oval vesicular nuclei with 1–3 prominent nucleoli. The cytoplasm may be abundant, sharply demarcated, and pale to "water–clear". Occasionally, large binucleate or multinucleate forms may simulate Reed–Sternberg cells. The atypia of the surrounding lymphocytes, however, clearly distinguishes these tumours from Hodgkin's disease. In the majority of cases there is an admixture of cytologically non-neoplastic epithelioid histiocytes, either in small aggregates or scattered singly throughout the tumour. A consistent feature of the series of Waldron *et al.* (1977) was a prominent proliferation of small vessels with endothelial hyperplasia. Studies of cellular suspensions have shown that the majority of the lymphoid cells, including both the small lymphocytes and the large atypical cells, form rosettes with sheep erythrocytes, indicating a neoplastic T-cell proliferation (Jaffe *et al.*, 1975; Waldron *et al.*, 1977). Mycosis fungoides and Sezary's syndrome, although not included in most classifications of non-Hodgkin's lymphomas, are also peripheral T-lymphocyte malignancies (Brouet *et al.*, 1973; Flandrin & Brouet, 1974; Lutzner *et al.*, 1975; Robinowitz *et al.*, 1976).

*Large cell lymphomas: "histiocytic", mixed lymphocytic–"histiocytic", and undifferentiated pleomorphic (non-Burkitt's)*

Diffuse lymphomas composed of large or medium-sized cells, or a mixture of the two have been categorized morphologically as "histiocytic", undifferentiated pleomorphic (non-Burkitt's), or mixed lymphocytic–"histiocytic", respectively. These tumours occur in both children and adults, but their incidence increases with age. Despite their propensity for extranodal presentations and relatively localized disease (Stage I or II) at the time of diagnosis (Chabner *et al.*, 1977) in general they pursue an aggressive clinical course with poor prognosis (Jones *et al.*, 1973). In this group of patients, however, recent trials of combination chemotheapy have been encouraging. If therapy does yield a well-documented complete clinical and pathological remission, there is a good chance for potential cure of the disease (Schein *et al.*, 1974, 1976; Berard *et al.*, 1976).

These tumours have marked cytological heterogeneity, and histologically are often difficult to classify reproducibly. Considerable effort has been directed to studying their immunological and cytochemical markers, which in published reports are also diverse (Peter & MacKenzie, 1974; Aisenberg & Long, 1975; Habeshaw & Stuart, 1975; Leech *et al.*, 1975; Morris & Davey, 1975; Bloomfield *et al.*, 1976; Brouet *et al.*, 1976; Davey *et al.*, 1976; Bloomfield *et al.*, 1977; Lawrence *et al.*, 1978; Lukes *et al.*, 1978*a*, *b*; Mann *et al.*, 1979; Pinkus *et al.*, 1979; Suchi *et al.*, 1979; Woda & Knowles, 1979). About 50–60% of cases have had characteristics of B lymphocytes, 5–15% have had markers of T lymphocytes, and only 5% have had features consistent with monocytes or true histiocytes. In about one third of the cases the cells have lacked detectable markers and have been termed "null" or undefined. We have recently completed an investigation of the surface markers and histochemical profile of 25 diffuse large-cell lymphomas. Morphologically 18 were classified as "histiocytic", 5 as undifferentiated pleomorphic, and 2 as mixed lymphocytic–"histiocytic". The malignant cells were studied in suspension for formation of spontaneous rosettes (E) with SRBC receptors for complement (EAC), receptors for cytophilic antibody (IgGEA) and surface immunoglobulins (SIg). Enzyme histochemical methods on frozen sections, and cytochemical reactions on cytocentrifuge preparations of selected cases, were used to detect the following hydrolytic enzymes: acid phosphatase (AP) with and without tartrate, alkaline phosphatase (ALP), β-glucuronidase (BG) and a battery of esterases (EST) including α-naphthyl acetate esterase (A-EST), α-naphthyl-butyrate esterase (B-EST), naphthol ASD chloroacetate esterase (ASDCL), and naphthyl ASD acetate esterase (NASDA) with and without inhibition by sodium fluoride (NaF). In Table VIII the immunological and histochemical features of these cases are summarized.

*B lymphocytic markers* were detected in 13/25 cases (52%). There was, however, marked variation in the number of mar-

TABLE VIII.—*Markers of diffuse large-cell lymphomas*

| B-cell | Source | Dx | SIg | Clon-ality | EAC | EA | E | B-EST | A-EST | AP | BG | TdT | Neoplastic cell markers | | | |
|---|---|---|---|---|---|---|---|---|---|---|---|---|---|---|---|---|
| 171* | LN | DH | 75 | ND | 14 | 19 | – | – | – | – | ND | – | SIg | EAC | EA | |
| 180* | LN | DH | 92 | ND | 43 | – | – | – | – | – | ND | – | SIg | EAC | | |
| 181* | LN | DH | NS | ND | 50 | ND | NS | – | – | – | ND | – | | EAC | | |
| 196* | LN | UND | 40 | ND | 80 | ND | – | – | – | – | ND | – | SIg | EAC | | |
| 251* | SPL | DH | 41 | Mk | 39 | 50 | – | – | – | – | – | – | SIg | EAC | EA | |
| | LN | DH | 45 | Mk | 64 | 41 | – | – | – | – | – | – | SIg | EAC | EA | |
| 271* | PB | DH | 85 | Mk | – | – | – | – | – | – | ++ | – | SIg | | | BG |
| 402* | LN | DH | 90 | ND | 9 | 9 | – | – | – | – | – | – | SIg | EAC | EA | |
| 134 | SPL | DH | 50 | ND | 50 | – | – | – | – | – | ND | – | SIg | EAC | | |
| 186 | BM | DH | 95 | Mk | – | 93 | – | – | + | + | ND | – | SIg | | EA | |
| 293 | LN | UND | 62 | k | 32 | – | – | – | – | – | – | – | SIg | EAC | | |
| 296 | INT | DH | 63 | Mk | 38 | – | – | – | + | + | + | – | SIg | EAC | | AP |
| 323 | LN | DH | 98 | Mk | 29 | – | – | – | – | – | – | – | SIg | EAC | | |
| 400 | PB | DM | 84 | Mk | – | 73 | – | – | ND | ND | ND | – | SIg | | EA | |
| T-cell | | | | | | | | | | | | | | | | |
| 174 | LN | DH | – | ND | NS | NS | 85 | + | ++ | ++ | ND | – | E | A-EST, AP | | |
| 397 | LN | UND | – | ND | – | – | 41 | – | – | ++ | ++ | – | E | AP, BG | | |
| 428 | PB | DM | – | ND | – | – | 55 | – | ND | ++ | ++ | – | E | AP, BG | | |
| 444 | LN | DH | – | ND | – | – | 61 | – | – | ++ | ++ | – | E | AP, BG | | |
| Hist. | | | | | | | | | | | | | | | | |
| 298 | Bone | DH | – | ND | 33 | 19 | – | + | ++ | ++ | ++ | – | EAC | EA | B-EST, A-EST, AP, BG | |
| Null | | | | | | | | | | | | | | | | |
| 47 | LN | DH | ND | ND | – | – | – | – | – | – | ND | ND | | | | |
| 91 | INT† | UND | ND | ND | – | – | – | – | – | – | ND | – | | | | |
| 210 | PB | DH | – | ND | – | – | – | – | ++ | ++ | ND | – | A-EST, AP | | | |
| 349 | ASC‡ | UND | – | ND | – | – | – | – | + | + | – | – | A-EST, AP, BG | | | |
| 389 | LN | DH | – | ND | – | – | – | – | – | – | – | +/– | | | | |
| 418 | LN | DH | – | ND | – | ND | – | – | – | – | – | – | | | | |
| 421 | LN | DH | – | – | – | – | – | – | + | + | ND | – | A-EST, AP | | | |

* Previous diagnosis of nodular (follicular) lymphoma.

† Intestine.

‡ Ascitic fluid.

Abbreviations:

Dx, pathological diagnosis; DH, diffuse "histiocytic"; UND, diffuse undifferentiated (non-Burkitt's); DM, diffuse mixed; NS, not satisfactory; ND, not done; B-EST, α-naphthyl butyrate esterase; A-EST, α-naphthyl acetate esterase; AP, acid phosphatase; BG, β-glucuronidase; TdT, terminal deoxynucleotidyl transferase.

kers identified, as well as in the percentages of neoplastic cells bearing particular markers. The most consistent marker was SIg, which was present in all thoroughly evaluated cases. In 8 cases assessed for individual heavy and light chains, IgM and $\kappa$ were identified. Surface-bound IgM has also been the most prevalent class of heavy chain in other B-lymphocytic neoplasms, including nodular (follicular) lymphoma (Aisenberg & Long, 1975; Leech *et al.*, 1975) chronic lymphocytic leukaemia (Preud'Homme & Seligmann, 1972) and Burkitt's tumour (Fialkow *et al.*, 1973; Mann *et al.*, 1976). These findings are not surprising since IgM is the immunoglobulin most frequently identified on B lymphocytes from normal peripheral blood (Aisenberg, 1973). One case bore only $\kappa$ light chains, without detectable heavy chains; however, this case was not evaluated for the presence of IgD. In none of these 13 B lymphocyte tumours was there significant hydrolytic-enzyme activity. Seven of the 25 large-cell lymphomas were from patients with a previous history of biopsy-proven nodular (follicular) lymphoma; these 7 *invariably* manifested B-cell markers. Such instances of "histological progression" with retention of markers appear to reflect heightened transformation and/or kinetic advantage within the neoplastic population. A similar phenomenon occurs in CML, with retention of the Ph′ chromosome during blastic transformation. Identical findings have been reported with other lymphoid neoplasms, most notably blastic transformation of CLL (Brouet *et al.*, 1974) and large-cell lymphomas supervening on CLL (Richter's syndrome) (Long & Aisenberg, 1975; Brouet *et al.*, 1976). In rare cases with immunological studies of both the antecedent well-differentiated proliferation and the blastic tumour, the membrane-bound immunoglobulins have shown persistence of the same light and heavy chains, a finding which supports the concept that both specimens belonged to a single neoplastic clone.

*T-lymphocyte markers* were demonstrated in 4/25 cases (16%). The malignant cells formed E rosettes and also had abundant granular reactivity for acid phosphatase and $\beta$-glucuronidase. Comparable enzymatic reactivity has been observed in other T-lymphocyte neoplasms, including Sezary's syndrome (Flandrin & Brouet, 1974) chronic lymphocytic leukaemia of T-cell type (Brouet *et al.*, 1975) and lymphoblastic lymphoma (Catovsky *et al.*, 1974; Stein *et al.*, 1976). Of particular interest was the fact that 1 of the 4 tumours with T-lymphocyte markers arose suddenly in a cervical lymph node of a 65-year-old white male with previously well-studied Sezary's syndrome. It was classified histologically as an undifferentiated lymphoma of non-Burkitt's type. The neoplastic cells, however, like the patient's Sezary's cells in earlier studies, manifested both T-cell surface characteristics and the ability to function as "helper" T cells for immunoblobulin synthesis by normal B lymphocytes *in vitro* (Lawrence *et al.*, 1978). As previously described with B-lymphocyte tumours, it thus appears that a malignancy of more differentiated T cells may undergo "histologic progression" and yet retain surface and functional properties characteristic of T lymphocytes.

In 1 of the 25 cases (4%) the markers were consistent with a *true histiocytic neoplasm*. The neoplastic cells were devoid of SIg but formed rosettes with both EAC and IgGEA. Additionally, they phagocytosed the bound erythrocytes, a phenomenon restricted in our experience to cells of the monocyte-macrophage series. They were rich in hydrolytic enzymes, with diffuse strong cytoplasmic activity for AP, A-EST, BG, and NASDA and weak staining for B-EST. This neoplasm arose in a 14-year-old boy and, unlike the other large-cell lymphomas in this series, appeared to be primary in bone (clavicle).

*No immunological markers* were detected in the remaining 7 cases (28%); 2 of them, however, were not evaluated for SIg. In 3 cases the malignant cells contained AP and A-EST; one of these was also studied for BG, with positive findings. The cytochemical staining pattern, however, was not diffuse as expected with cells of the monocyte–macrophage series. Instead, the enzymatic reaction was manifested as discrete punctate dots, often restricted to the Golgi zone. This pattern of reactivity occurs in lymphocytes, both normal and neoplastic. It has been reported in T-cell tumours, but these cases lacked T-cell markers. All 3 of these cases were also studied for and lacked terminal deoxynucleotidyl transferase (TdT) (Bollum, 1979). All 3 had plasmacytoid features, and 2 examined in the electron microscope had moderate to abundant rough endoplasmic reticulum (Fisher *et al.*, 1976). Strong AP activity can be demonstrated in mature plasma cells and the neoplastic cells of multiple myeloma. Unlike such cells, however, the malignant cells of these lymphomas were devoid of demonstrable cytoplasmic immunoglobulin. In one of the "null" cases [389] attempts were made to detect TdT, using both a biochemical assay and indirect immunofluorescence (Bollum, 1979). Although the biochemical assay was positive, immunofluorescence failed to confirm this. Since, in a series of more than 50 cases, this is the sole instance in which immunofluorescence failed to confirm the biochemical assay, the positivity of this case for TdT remains dubious.

*Undifferentiated, Burkitt's type*

Although there are clinical differences between patients with Burkitt's lymphoma in endemic areas of East Africa and those in non-endemic regions of the world, the tumours are histologically identical (Banks *et al.*, 1975). They are composed of a strikingly uniform population of cytologically "primitive" cells with characteristic features in imprints and technically optimal histological sections. They are 10–25 $\mu$m in diameter and have round-to-oval nuclei with 2–5 basophilic nucleoli. The chromatin is coarsely reticulated and irregularly distributed within a rather clear parachromatin. The cytoplasm is rich in ribosomes and consequently amphophilic, with a hue similar to that of normal plasma cells. Special stains reveal intense cytoplasmic pyroninophilia, sometimes highlighting clear cytoplasmic vacuoles. In frozen sections or air-dried imprints some of these vacuoles contain demonstrable neutral lipids. Mitoses are abundant ($\sim 4\%$ of the cells) and nuclear pyknosis and karyorrhexis are usually conspicuous. Characteristically but not invariably, tingible-body macrophages are scattered throughout the tumour with a resultant "starry-sky" appearance. These features all reflect the high growth fraction and rapid kinetics characteristic of Burkitt's lymphoma (Braylan *et al.*, 1978). The cytological uniformity mirrors the kinetic uniformity of the neoplastic cells. In untreated cases the disease is very aggressive and usually rapidly fatal, but gratifying results have been obtained with modern intensive chemotherapy in both African and American patients (Ziegler, 1977).

Immunologically the neoplastic B cells from both patient populations are identical. They characteristically have abundant monoclonal SIg, usually of the IgM class, but inconstantly manifest receptors for complement (Fialkow *et al.*, 1973; Mann *et al.*, 1976). Within the cells there is little or no demonstrable activity of hydrolytic enzymes. Lukes & Collins (1974) have likened the cells of Burkitt's lymphoma cytologically to small non-cleaved cells of germinal centres. To pursue this suggestion, we thoroughly reviewed all the histopathological sections from 47 biopsy and 17 necropsy specimens, specifically to assess patterns of neoplastic proliferation in lymph nodes, spleens, and Peyer's patches. In 10 biopsy and 2 necropsy specimens, there was selective involvement of germinal centres by Burkitt's tumour. Data both from human cases and experiments in animals support the concept of a relationship between the cells of Burkitt's tumour and some B lymphocytes of normal germinal centres (Mann *et al.*, 1976; Mann & Berard, 1977). Nevertheless, the site of origin of Burkitt's lymphoma in man has yet to be conclusively demonstrated.

*Lymphoblastic*

Lymphoblastic lymphoma is now recognized as a specific clinicopathological entity (Nathwani *et al.*, 1976; Jaffe & Berard, 1978) formerly included in the generic group of diffuse poorly differentiated lymphocytic lymphomas. The disease occurs most frequently in adolescents though there is a broad age range. Males are affected much more often than females, and ~50% of the patients have mediastinal masses at presentation. The clinical course is rapidly progressive, with spread of the neoplastic cells to marrow, peripheral blood, and cerebrospinal fluid. Historically the prognosis has been dismal, with a median survival of only 8 months in one large series (Nathwani *et al.*, 1976). With modern intensive therapy, however, of the type used for acute lymphoblastic leukaemia, the outlook has improved markedly (Weinstein *et al.*, 1979). The tumours are composed of relatively monomorphic cells with sparse cytoplasm and round or convoluted nuclei. Chromatin is delicately stippled and evenly distributed, and nucleoli are usually small and inconspicuous. In a majority of cases one can identify, with a 100X oil-immersion objective, variably numerous cells with convoluted nuclear infoldings and lobulations. Mitotic figures are numerous and may be accompanied by a "starry-sky" pattern of interspersed macrophages identical to those of Burkitt's tumour.

The frequent association of lymphoblastic lymphoma with an anterior mediastinal mass at presentation led early on to the suggestion of a link between this tumour and the thymus gland (Webster, 1961). Moreover, the observation of selective neoplastic infiltration of the paracortical T cell regions of partially involved lymph nodes strengthened the hypothesis that this lymphoma was closely related to T lymphocytes. In view of these indirect observations it is not surprising that T-cell markers have been identified in most cases. The malignant cells have in particular the surface, cytochemical and biochemical features of immature thymocytes (Kaplan *et al.*, 1974; Gatien *et al.*, 1975; Coccia *et al.*, 1976; Jaffe *et al.*, 1976; Stein *et al.*, 1976; Donlon *et al.*, 1977; Stein & Muller-Hermelink, 1977; Kersey *et al.*, 1978; Kung *et al.*, 1978; Lukes *et al.*, 1978*a*, *b*; Bollum, 1979; Long *et al.*, 1979). The marked propensity for progression to leukaemia establishes a continuum between these tumours and a subset of acute lymphoblastic leukaemia (ALL), *i.e.*, the 25% of cases of ALL with T-cell markers (Kersey *et al.*, 1973; Sen & Borella, 1975; Tsukimoto *et al.*, 1976).

The immunological, cytochemical and biochemical features of 12 cases studied in our laboratory are summarized in Table IX. Nine of 12 (75%) were males and all but one were aged 30 years or less. At presentation 10/12 (83%) had anterior mediastinal masses. In only 6/12 (50%) did the malignant cells manifest T markers as defined by formation of E rosettes. Perhaps if they had been studied for T-cell-associated heteroantigens, more of these cases would have been provably T cell in nature. Complement receptors were demonstrated in 5 cases (42%) and, in 3 of these, sheep-erythrocyte receptors and complement receptors coexisted. Four cases (33%) had no demonstrable markers. Cytochemical studies for acid phosphatase (AP) were performed on 8 cases. Some degree of AP activity was present in all but the reaction was strong and sharply localized only in cases with formation of E rosettes by the neoplastic cells. Intense punctate staining for AP has also been reported in T-cell ALL (Catovsky *et al.*, 1974). In our series of lymphoblastic lymphomas, cases which did not form E rosettes tended to manifest AP activity as a diffuse multigranular reaction. All cases studied were positive for TdT (Donlon *et al.*, 1977; Bollum, 1979). The heterogeneity of markers in this series confirms our earlier experience (Jaffe *et al.*, 1976). Conceptually these tumours seem to recapitulate different stages in the

TABLE IX.—*Markers of lymphoblastic lymphomas*

| Case No. | Age/sex | Med. mass | Source | E* | EAC | SIg | AP | TdT |
|---|---|---|---|---|---|---|---|---|
| 87 | 12F | + | PB | 7 | 85† | 8 | ND | ND |
| 157 | 23M | + | PB | 10 | 9 | 8 | ND | ND |
| 162 | 18M | + | BM | 3 | 4 | 0 | ND | ND |
| 166 | 22M | + | PB | 80† | 70† | 0 | ND | ND |
| 240 | 19M | + | LN | 7 | 30† | 0 | M‡ | + |
| 306 | 60M | – | Skin | 20† | 9 | 0 | ND | + |
| | | – | PB | 6 | 3 | ND | M‡ | – |
| 312 | 18M | + | PF | 32† | 34† | 1 | P‡ | + |
| 344 | 30F | + | PF | 23† | 16† | 0 | P‡ | + |
| 376 | 23M | + | PF | 63† | NS | 0 | P‡ | + |
| 394 | 22M | + | LN | 70† | 1 | 2 | P‡ | + |
| 405 | 8F | – | Tibia | 0 | ND | 1 | M‡ | + |
| 485 | 25M | + | BM | 3 | 4 | 35 | M‡ | ND |

* Results are expressed as % positive cells.
† Indicates marker identified on neoplastic cells.
‡ M = Multigranular reaction product; P = Punctate perinuclear reaction product.
Abbreviations: PF, pleural fluid; Med. mass, mediastinal mass; ND, not determined; NS, not satisfactory; AP, acid phosphatase; TdT ,terminal deoxynucleotidyl transferase.

maturation of thymic lymphoblasts. As with foetal thymocytes (Gatien *et al.*, 1975; Stein & Muller-Hermelink, 1977), complement receptors seem to be present on the most primitive cells and are lost as the cells differentiate or "mature". TdT is present in all cases but the development of sharply localized acid phosphatase activity seems to correlate with appearance of the ability to form E rosettes.

*Hodgkin's disease*

Despite impressive clinical advances in the diagnosis, staging, and therapy of patients with H.D. there is still no definitive evidence for the nature of the neoplastic cells in this condition (Long, 1979). Because of the complex composition of the cellular proliferation, there are unavoidable difficulties in isolating and characterizing the neoplastic cells. In most non-Hodgkin's lymphomas and leukaemias, the malignant populations are fairly homogeneous and studies of cellular suspensions may readily reveal their nature. In contrast, H.D. is composed of individual malignant cells distributed amidst presumed reactive elements which usually comprise the bulk of the tumefaction. This interaction of reactive and malignant cells has been confirmed by the documentation of both euploid and aneuploid populations in H.D. lesions (Peckham & Cooper, 1969). For these reasons, aggregated numerical data gleaned from cellular suspensions of involved tissues do not necessarily reflect specific markers on the neoplastic cells. Such mixed populations can be assessed accurately only with a combination of morphological, immunological, and cytochemical techniques. Meaningful studies are further hampered by the considerable difficulties encountered in attempting to harvest adequate numbers of malignant cells from H.D. tissue, perhaps due to the fragile nature of such cells and/or the fibrosis common in these lesions (Leech, 1973; Braylan *et al.*, 1974). In an attempt to circumvent these problems, some investigators have recently tried to characterize the neoplastic cells either *in situ* (*i.e.*, in frozen or paraffin-embedded tissue sections) or in long-term tissue culture.

Although classically interpreted on morphological grounds as probably deriving from "reticulum cells" or histiocytes (Rappaport, 1966) Reed–Sternberg cells in early histochemical studies lacked the enzymatic apparatus of cells of the monocyte–macrophage series (Dorfman, 1961). With the realization in recent years that, in response to mitogens or specific antigens, B and T lymphocytes can transform to large cells with vesicular nuclei, prominent nucleoli, and deeply basophilic cytoplasm, the suggestion has been made that the malignant cells of H.D. may actually derive from transformed lymphocytes rather than histiocytes. Distinct similarities between Reed–Sternberg cells and transformed lymphocytes have been demonstrated ultrastructurally (Glick *et al.*, 1976).

H.D. shows strong evidence of a derangement of the T-cell limb of the immune system (Long, 1979). Untreated patients with newly diagnosed and even localized disease may exhibit functionally deficient T-cell-mediated immune responses, such as impaired delayed hypersensitivity (Young *et al.*, 1973) prolonged retention of cutaneous grafts (Kelly *et al.*, 1960), and increased susceptibility to certain infectious organisms (Casazza *et al.*, 1966). Levels of E-rosette-forming cells in the peripheral blood are often reduced, perhaps due to circulating soluble factors. The defect in formation of E rosettes can be abolished by incubation in tissue culture medium with 20% foetal calf serum; it can be reinduced by incubation in sera from H.D. patients. Such sera, however, do not exert this effect on T lymphocytes from control patients (Fuks *et al.*, 1976). Also linking H.D. to the T-cell system is the often striking preferential localization of tumour within thymic-dependent paracortical regions of partially involved lymph nodes. On the basis of these indirect clues, some investigators have suggested that the Reed–Sternberg cell is a transformed T lymphocyte, perhaps antigenically altered by viral infection (Order & Hellman, 1972; DeVita, 1973; Biniaminov & Ramot, 1974). They have hypothesized that H.D. represents a "civil war" of interaction between antigenically altered T lymphocytes and normal uninfected lymphocytes. Although enticing, this theory is devoid of any firm evidence. Published immunological studies of Reed–Sternberg cells in suspension have failed to demonstrate any markers of T lymphocytes (Boecker *et al.*, 1975; Kay & Kadin, 1975; Schmitt *et al.*, 1977; Stuart *et al.*, 1977).

The finding of immunoglobulins, both within (Garvin *et al.*, 1974; Taylor, 1974, 1976; Payne *et al.*, 1976) and on the surface (Kadin *et al.*, 1974; Boecker *et al.*, 1975) of Reed–Sternberg cells, has prompted the alternative notion that they are neoplastic B lymphocytes. Whilst isolated reports exist of monoclonal cytoplasmic immunoglobulin, most cases studied by either immunoperoxidase techniques or immunofluorescence have contained polyclonal immunoglobulins (Garvin *et al.*, 1974; Taylor, 1974, 1976; Kadin *et al.*, 1978; Poppema *et al.*, 1978). Certainly the mere presence of immunoglobulin, without evidence of endogenous synthesis, does not identify a cell as a B lymphocyte. Receptors for the Fc portion of IgG have been demonstrated on Reed–Sternberg cells (Jaffe *et al.*, 1974b; Payne *et al.*, 1976) and recent *in vitro* studies have directly documented the internalization of exogenous IgG and phagocytosis of immune complexes by viable Reed–Sternberg cells (Kadin *et al.*, 1978). Additionally, patients with active H.D. have been shown to have circulating immune complexes (Amlot *et al.*, 1976). These complexes appear to bind specifically to cell lines established in long-term culture from spleens involved by H.D. (Long *et al.*, 1977*a*). Analogous binding may occur *in vivo* and account for the presence of immunoglobulin on the surface of and within the malignant cells. There is thus no definitive proof of a B-cell origin of Reed–Sternberg cells.

*In vitro* studies of viable Reed–Sternberg cells have suggested derivation from macrophages (Kadin *et al.*, 1978). Support for this possibility has come from 3 laboratories independently studying long-term cultures of cells established *in vitro* from H.D. tumors (Kaplan & Gartner, 1977; Long *et al.*, 1977*b*; Roberts *et al.*, 1978). The cultured cells cytologically resemble Reed–Sternberg cells and their mononuclear counterparts. They also fulfil criteria of malignancy by being aneuploid and heterotransplantable. All the cell lines lack SIg, fail to form E rosettes, and manifest to variable degrees cytochemical and functional markers most consistent with macrophages. These findings should be confirmed and extended, with particular emphasis on proving that the cultured cells really are *in vitro* descendants of the malignant cells of Hodgkin's disease.

### REFERENCES

AISENBERG, A. C. (1973) Malignant lymphoma. *N. Engl. J. Med.*, **288**, 883.

AISENBERG, A. C., BLOCH, J. K. & LONG, J. C. (1973) Cell surface immunoglobulins in chronic lymphocytic and allied disorders. *Am. J. Med.*, **55**, 184.

AISENBERG, A. C. & LONG, J. C. (1975) Lymphocyte surface characteristics in malignant lymphoma. *Am. J. Med.*, **58**, 300.

AISENBERG, A. C. & WILKES, B. (1976) Lymphosarcoma cell leukaemia: The contribution of cell surface study to diagnosis. *Blood*, **48**, 707.

AMLOT, P. L., SLANEY, J. M. & WILLIAMS, B. D. (1976) Circulating immune complexes and symptons in Hodgkin's disease. *Lancet*, i, 449.

BANKS, P. M., ARSENEAU, J. C., GRALNICK, H. R., CANELLOS, G. P., DEVITA, V. T. & BERARD, C. W. (1975) American Burkitt's lymphoma: A clinicopathologic study of 30 cases. II. Pathologic correlations. *Am. J. Med.*, **58**, 322.

BERARD, C. W., GALLO, R. C., JAFFE, E. S., GREEN, I. & DEVITA, V. T. (1976) Current concepts of leukemia and lymphoma: Etiology, pathogenesis, and therapy. *Ann. Intern. Med.*, **85**, 351.

BERARD, C. W., JAFFE, E. S., BRAYLAN, R. C., MANN, R. B. & NANBA, K. (1978) Immunologic aspects and pathology of the malignant lymphomas. *Cancer*, **42**, 911.

BINIAMINOV, M. & RAMOT, B. (1974) Possible T-lymphocyte origin of Reed-Sternberg cells. *Lancet*, i, 368.

BLOOMFIELD, C. D., KERSEY, J. H., BRUNNING, R. D. & GAJL-PECZALSKA, K. J. (1976) Prognostic significance of lymphocyte surface markers in adult non-Hodgkin's malignant lymphoma. *Lancet*, ii, 1330.

BLOOMFIELD, C. D., KERSEY, J. H., BRUNNING, R. D. & GAJL-PECZALSKA, K. J. (1977) Prognostic significance of lymphocytic surface markers and histology in adult non-Hodgkin's lymphoma. *Cancer Treat. Rep.*, **61**, 963.

BOECKER, W. R., HOSSFELD, D. K., GALLMEIER, W. M. & SCHMIDT, C. G. (1975) Clonal growth of Hodgkin cells. *Nature*, **258**, 235.

BOLLUM, F. J. (1979) Terminal deoxynucleotidyl transferase as a hematopoietic cell marker. *Blood*, **54**, 1203.

BRAGANZA, C. M., STATHOPOULOUS, G., DAVIES, A. J. S. & 4 others (1975) Lymphocyte: Erythrocyte (L.E.) rosettes as indicators of the heterogeneity of lymphocytes in a variety of mammalian species. *Cell.*, **4**, 103.

BRAYLAN, R. C., FOWLKES, B. J., JAFFE, E. S., SANDERS, S. K., BERARD, C. W. & HERMAN, C. J. (1978) Cell volumes and DNA distributions of normal and neoplastic human lymphoid cells. *Cancer*, **41**, 201.

BRAYLAN, R. C., JAFFE, E. S. & BERARD, C. W. (1974) Surface characteristics of Hodgkin's lymphoma cells. *Lancet*, ii, 1328.

BRAYLAN, R. C., JAFFE, E. S., BURBACH, J. W., FRANK, M. M., JOHNSON, R. C. & BERARD, C. W. (1976) Similarities of surface characteristics of neoplastic well-differentiated lymphocytes from solid tissues and from peripheral blood. *Cancer Res.*, **36**, 1619.

BRAYLAN, R. C., LONG, J. C., JAFFE, E. S., GRECO, F. A., ORR, S. L. & BERARD, C. W. (1977) Lymphoid neoplasm obscured by concomitant extensive epithelioid granulomas. Report of three cases with similar clinicopathologic features. *Cancer*, **39**, 1146.

BROUET, J. C., FLANDRIN, G., SASPORTES, M., PREUD'HOMME, J. L. & SELIGMANN, M. (1975) Chronic lymphocytic leukemia of T-cell origin. Immunological and clinical evaluation in eleven patients. *Lancet*, ii, 890.

BROUET, J. C., FLANDRIN, G. & SELIGMANN, H. (1973) Indications of the thymus derived nature of the proliferating cells in six patients with Sezary's syndrome. *N. Engl. J. Med.*, **289**, 341.

BROUET, J. C., LABAUME, S. & SELIGMANN, M. (1975) Evaluation of T and B lymphocyte membrane markers in human non-Hodgkin malignant lymphomata. *Br. J. Cancer*, **31** (Suppl. 2), 131.

BROUET, J. C., PREUD'HOMME, J. L., FLANDRIN, G., CHELLOUL, N. & SELIGMANN, M. (1976) Brief communication: Membrane markers in "histiocytic" lymphomas (reticulum cell sarcomas). *J. Natl Cancer Inst.*, **56**, 631.

BROUET, J. C., PREUD'HOMME, J. & SELIGMANN, M. (1974) Blast cells with monoclonal surface immunoglobulin in two cases of acute blast crisis supervening in chronic lymphocytic leukemia. *Br. Med. J.*, iv, 23.

CASAZZA, A. R., DUVALL, C. P. & CARBONE, P. P. (1966) Summary of infectious complications occurring in patients with Hodgkin's disease. *Cancer Res.*, **26**, 1290.

CATOVSKY, D., GALETTO, J., OKOS, A., MILANI, E. & GALTON, D. A. G. (1974) Cytochemical profile of B and T leukaemic lymphocytes with special reference to acute lymphoblastic leukaemia. *J. Clin. Pathol.*, **27**, 767.

CHABNER, B. A., JOHNSON, R. E., DEVITA, V. T. & 4 others (1977) Sequential staging in non-Hodgkin's lymphoma. *Cancer Treat. Rep.*, **61**, 993.

COCCIA, P. F., KERSEY, J. H., GAJL-PECZALSKA, K. J., KRIVIT, W. & NESBIT, M. E. (1976) Prognostic significance of surface marker analysis in childhood non-Hodgkin's lymphoproliferative malignancies. *Am. J. Hematol.*, **1**, 405.

CRADDOCK, C. G., LONGMIRE, R. & MCMILLAN, R. (1971) Lymphocytes and the immune response (second of two parts). *N. Engl. J. Med.*, **285**, 378.

CULLEN, M. H., LISTER, T. A., BREARLEY, R. L., SHAND, W. S. & STANSFELD, A. G. (1979) Histological transformation of non-Hodgkin's lymphoma. A prospective study. *Cancer*, **44**, 645.

DAVEY, F. R., GOLDBERG, J., STOCKMAN, J. & GOTTLIEB, A. J. (1976) Immunologic and cytochemical cell markers in non-Hodgkin's lymphomas. *Lab. Invest.*, **35**, 430.

DEVITA, V. T. (1973) Lymphocyte reactivity in Hodgkin's disease. A lymphocyte civil war. *N. Engl. J. Med.*, **289**, 801.

DONLON, J. A., JAFFE, E. S. & BRAYLAN, R. C. (1977) Terminal deoxynucleotidyl transferase activity in malignant lymphomas. *N. Engl. J. Med.*, **297**, 461.

DORFMAN, R. F. (1961) Enzyme histochemistry of the cells in Hodgkin's disease and allied disorders. *Nature*, **190**, 925.

FIALKOW, P. J., KLEIN, E., KLEIN, G., CLIFFORD, P. & SINGH, S. (1973) Immunoglobulin and glucose-6-phosphate dehydrogenase as markers of cellular origin in Burkitt lymphoma. *J. Exp. Med.*, **138**. 89.

FISHER, R. I., JAFFE, E. S. & BRAYLAN, R. C. (1976) Immunoblastic lymphadenopathy: evolution into a malignant lymphoma with plasmacytoid features. *Am. J. Med.*, **61**, 553.

FLANDRIN, G. & BROUET, J. C. (1974) The Sezary cell: Cytologic, cytochemical and immunologic features. *Mayo Clin. Proc.*, **49**, 575.

FU, S. M., CHIORAZZI, N., KUNKEL, H. G., HALPER, J. P. & HARRIS, S. R. (1978) Induction of *in vitro* differentiation and immunoglobulin synthesis of human leukemic B lymphocytes. *J. Exp. Med.*, **148**, 1570.

FU, S. M., WINCHESTER, R. J., FEIZI, T., WALZER, P. D. & KUNKEL, H. G. (1974) Idiotypic specificity of surface immunoglobulin and the maturation of leukemic bone marrow derived lymphocytes. *Proc. Natl Acad. Sci. U.S.A.*, **71**, 4487.

FUKS, Z., STROBER, S. & KAPLAN, H. S. (1976) Interaction between serum factors and T lymphocytes in Hodgkin's disease. *N. Engl. J. Med.*, **295**, 1273.

GARVIN, A. J., SPICER, S. S., PARMLEY, R. T. & MUNSTER, A. M. (1974) Immunohistochemical demonstration of IgG in Reed-Sternberg and other cells in Hodgkin's disease. *J. Exp. Med.*, **139**, 1077.

GATIEN, J. G., SCHNEEBERGER, E. E. & MERLER, E. (1975) Analysis of human thymocyte subpopulations using discontinuous gradients of albumin: Precursor lymphocytes in human thymus. *Eur. J. Immunol.*, **5**, 312.

GLICK, A. D., LEECH, J. H., FLEXNER, J. M. & COLLINS, R. D. (1976) Ultrastructural study of Reed-Sternberg cells: Comparison with transformed lymphocytes and histiocytes. *Am. J. Pathol.*, **85**, 195.

HABESHAW, J. A. & STUART, A. E. (1975) Cell receptor studies on seven cases of diffuse histiocytic malignant lymphoma (reticulum cell sarcoma). *J. Clin. Pathol.*, **28**, 289.

JAFFE, E. S. & BERARD, C. W. (1978) Lymphoblastic lymphoma: A term rekindled with new precision. *Ann. Intern. Med.*, **89**, 415.

JAFFE, E. S., BRAYLAN, R. C., FRANK, M. M., GREEN, I. & BERARD, C. W. (1976) Heterogeneity of immunologic markers and surface morphology in childhood lymphoblastic lymphoma. *Blood*, **48**, 213.

JAFFE, E. S., SHEVACH, E. M., FRANK, M. M., BERARD, C. W. & GREEN, I. (1974*a*) Nodular lymphoma: Evidence for origin from follicular B lymphocytes. *N. Engl. J. Med.*, **290**, 813.

JAFFE, E. S., SHEVACH, E. M., FRANK, M. M., BERARD, C. W. & GREEN, I. (1974*b*) The immunological identification of malignant lymphoreticular cells. *J. Reticuloendoth. Soc.*, **15**, 76a.

JAFFE, E. S., SHEVACH, E. M., SUSSMAN, E. H., FRANK, M., GREEN, I. & BERARD, C. W. (1975) Membrane receptor sites for the identification of lymphoreticular cells in benign and malignant conditions. *Br. J. Cancer*, **31** (Suppl. 2), 107.

JONES, S. E., FUKS, Z., BULL, M., KADEN, M. E. & 4 others (1973) Non-Hodgkin's lymphomas. IV. Clinicopathologic correlation in 405 cases. *Cancer*, **31**, 806.

KADIN, M. E., NEWCOM, S. R., GOLD, S. B. & STITES, D. P. (1974) Origin of Hodgkin's cell. *Lancet*, ii, 167.

KADIN, M. E., STITES, D. P., LEVY, R. & WARNKE, R. (1978) Exogenous immunoglobulin and the macrophage origin of Reed–Sternberg cells in Hodgkin's disease. *N. Engl. J. Med.*, **299**, 1208.

KAPLAN, H. S. & GARTNER, S. (1977) "Sternberg–Reed" giant cells of Hodgkin's disease: Cultivation *in vitro*, heterotransplantation, and characterization as neoplastic macrophages. *Int. J. Cancer*, **19**, 511.

KAPLAN, J., MASTRANGELO, R. & PETERSON, W. D. (1974) Childhood lymphoblastic lymphoma, a cancer of thymus-derived lymphocytes. *Cancer Res.*, **34**, 521.

KAY, M. M. B. & KADIN, M. (1975) Surface characteristics of Hodgkin's cells. *Lancet*, i, 748.

KELLY, W. D., LAMB, D. L., VARCO, R. L. & GOOD, R. A. (1960) An investigation of Hodgkin's disease with respect to the problem of homotransplantation. *Ann. N.Y. Acad. Sci.*, **87**, 187.

KERSEY, J. H., GAJL-PECZALSKA, K. J., COCCIA, P. F. & NESBIT, M. E. (1978) The nature of childhood leukemia and lymphoma. *Am. J. Pathol.*, **90**, 487.

KERSEY, J. H., SABAD, Z., GAJL-PECZALSKA, K., HALLGREN, H. M., YUNIS, E. J. & NESBIT, M. E. (1973) Acute lymphoblastic leukemia cells with T (Thymus-Derived) lymphocyte markers. *Science*, **182**, 1355.

KOJIMA, M., IMAI, Y. & MORI, N. (1973) A concept of follicular lymphoma—A proposal for the existence of a neoplasm originating from the germinal center. In *Gann Monograph on Cancer Research*, **15**. Tokyo: University Press. p. 195.

KUNG, P. C., LONG, J. C., MCCAFFREY, R. P., RATLIFF, R. L., HARRISON, T. A. & BALTIMORE, D. (1978) Terminal deoxynucleotidyl transferase in the diagnosis of leukemia and malignant lymphoma. *Am. J. Med.*, **64**, 788.

LAWRENCE, E. C., BRODER, S., JAFFE, E. S. & 4 others (1978) Evolution of a lymphoma with helper T-cell characteristics in Sezary syndrome. *Blood*, **52**, 481.

LEECH, J. (1973) Immunoglobulin-positive Reed–Sternberg cells in Hodgkin's disease. *Lancet*, ii, 265.

LEECH, J. H., GLICK, A. D., WALDRON, J. A., FLEXNER, J. M., HORN, R. G. & COLLINS, R. D. (1975) Malignant lymphomas of follicular center cell origin in man. I. Immunologic studies. *J. Natl Cancer Inst.*, **54**, 11.

LENNERT, K. (1973) The follicular lymphoma—A tumor of the germinal centers. In *Gann Monograph on Cancer Research*. **15**. Tokyo: University Press. p. 217.

LEVINE, G. D. & DORFMAN, R. F. (1975) Nodular lymphoma: An ultrastructural study of its relationship to germinal centers and a correlation of light and electron microscopic findings. *Cancer*, **35**, 148.

LOGUE, G. L. & COHEN, H. J. (1977) Human lymphocyte complement receptors. Quantitative requirements for C3 of normal and chronic lymphocytic leukemia lymphocytes. *J. Clin. Invest*, **60**, 1159.

LOHRMAN, H. P. & NOVIKOVS, L. (1974) Rosette formation between human T lymphocytes and unsensitized Rhesus monkey erythrocytes. *Clin. Immunol. Immunopathol.*, **3**, 99.

LONG, J. C. (1979) The immunopathology of Hodgkin's disease. *Clin. Haematol.*, **8**, 531.

LONG, J. C. & AISENBERG, A. C. (1975) Richter's syndrome. A terminal complication of chronic lymphocytic leukemia with distinct clinicopathologic features. *Am. J. Clin. Pathol.*, **63**, 786.

LONG, J. C., HALL, C. L., BROWN, C. A., STAMATOS, C., WEITZMAN, S. A. & CAREY, K. (1977*a*) Binding of soluble immune complexes in serum of patients with Hodgkin's disease to tissue cultures derived from the tumor. *N. Engl. J. Med.*, **297**, 295.

LONG, J. C., MCCAFFREY, R. P., AISENBERG, A. C., MARKS, S. M. & KUNG, P. C. (1979) Terminal deoxynucleotidyl transferase positive lymphoblastic lymphoma. A study of 15 cases. *Cancer*, **44**, 2127.

LONG, J. C., ZAMECNIK, P. C., AISENBERG, A. C. & ATKINS, L. (1977*b*) Tissue culture studies in Hodgkin's disease. Morphologic, cytogenic, cell surface, and enzymatic properties of cultures derived from splenic tumors. *J. Exp. Med.*, **145**, 1484.

LUKES, R. J. & BUTLER, J. J. (1966) The pathology and nomenclature of Hodgkin's disease. *Cancer* Res., **26**, 1063.

LUKES, R. J., BUTLER, J. J. & HICKS, E. B. (1966*a*) Natural history of Hodgkin's disease as related to its pathologic picture. *Cancer*, **19**, 317.

LUKES, R. J. & COLLINS, R. D. (1974) Immunologic characterization of human malignant lymphomas. *Cancer*, **34**, 1488.

LUKES, R. J., CRAVER, L. F., HALL, T. C., RAPPAPORT, H. & RUBEN, P. (1966*b*) Report of the Nomenclature Committee. *Cancer Res.*, **26**, 1311.

LUKES, R. J., PARKER, J. W., TAYLOR, C. R., TINDLE, B. H., CRAMER, A. D. & LINCOLN, T. L. (1978*a*) Immunologic approach to non-Hodgkin's lymphomas and related leukemias. Analysis of the results of multiparameter studies of 425 cases. *Sem. Hematol.*, **15**, 322.

LUKES, R. J., TAYLOR, C. R., PARKER, J. W., LINCOLN, T. L., PATTENGALE, P. K. & TINDLE, B. H. (1978*b*) A morphologic and immunologic surface marker study of 299 cases of non-Hodgkin lymphomas and related leukemias. *Am. J. Pathol.*, **90**, 461.

LUTZNER, M., EDELSON, R., SCHEIN, P., GREEN, I., KIRKPATRICK, C. & AHMED, A. (1975) Cutaneous T-cell lymphomas. The Sezary syndrome, mycosis fungoides, and related disorders. *Ann. Intern. Med.*, **83**, 534.

MANN, R. B. & BERARD, C. W. (1977) Burkitt's tumor: Lessons from mice, monkeys, and man. *Lancet*, ii, 84.

MANN, R. B., JAFFE, E. S. & BERARD, C. W. (1979) Malignant lymphomas—a conceptual understanding of morphologic diversity. *Am. J. Pathol.*, **94**, 103.

MANN, R. B., JAFFE, E. S., BRAYLAN, R. C. & 4 others (1976) Non-endemic Burkitt's lymphoma: A B cell tumor related to germinal centers. *N. Engl. J. Med.*, **295**, 685.

MORRIS, M. W. & DAVEY, F. R. (1975) Immunologic and cytochemical properties of histiocytic and mixed histiocytic-lymphocytic lymphomas. *Am. J. Clin. Pathol.*, **63**, 403.

NANBA, K., JAFFE, E. S., BRAYLAN, R. C., SOBAN, E. J. & BERARD, C. W. (1977) Alkaline phosphatase-positive malignant lymphoma. A subtype of B-cell lymphomas. *Am. J. Clin. Pathol.*, **68**, 535.

NATHWANI, B. N., KIM, H. & RAPPAPORT, H. (1976) Malignant lymphoma, lymphoblastic. *Cancer*, **38**, 964.

O'CONNELL, C. J. (1973) Detection of lymphocyte rosettes in tissues. *N. Engl. J. Med.*, **289**, 1312.

ORDER, S. E. & HELLMAN, S. (1972) Pathogenesis of Hodgkin's disease. *Lancet*, i, 571.

PANGALIS, G. A., NATHWANI, B. N. & RAPPAPORT, H. (1977) Malignant lymphoma, well differentiated lymphocytic. Its relationship with chronic lymphocytic leukemia and macroglobulinemia of waldenstrom. *Cancer*, **39**, 999.

PAYNE, S. V., JONES, D. B., HAEGERT, D. C., SMITH, J. L. & WRIGHT, D. H. (1976) T and B lymphocytes and Reed–Sternberg cells in Hodgkin's disease lymph nodes and spleens. *Clin. Exp. Immunol.*, **24**, 280.

PECKHAM, M. J. & COOPER, E. H. (1969) Proliferation characteristics of the various classes of cells in Hodgkin's disease. *Cancer*, **24**, 135.

PETER, C. R. & MACKENZIE, M. P. (1974) T or B cell origin of some non-Hodgkin's lymphomas. *Lancet*, ii, 686.

PINKUS, G. S., SAID, J. W. & HARGREAVES, H. (1979) Malignant lymphoma, T-cell type. A distinct morphologic variant with large multilobated nuclei, with a report of four cases. *Am. J. Clin. Pathol.*, **72**, 540.

POPPEMA, S., ELEMA, J. D. & HALIE, M. R. (1978) The significance of intracytoplasmic proteins in Reed–Sternberg cells. *Cancer*, **42**, 1793.

POTVIN, C., TARPLEY, J. L. & CHRETEIN, P. (1975) Thymus-derived lymphocytes in patients with solid malignancies. *Clin. Immunol. Immunopathol.*, **3**, 476.

PREUD'HOMME, J. L. & SELIGMANN, M. (1972) Surface bound immunoglobulins as a cell marker in human lymphoproliferative diseases. *Blood*, **40**, 777.

QAZI, R., AISENBERG, A. C. & LONG, J. C. (1976) The natural history of nodular lymphoma. *Cancer*, **37**, 1923.

RAPPAPORT, H. (1966) Tumors of the hematopoietic system. In *Atlas of Tumor Pathology*, Sec. III, Fasc. 8. Washington, D.C.: Armed Forces Institute of Pathology.

RISDALL, R., HOPPE, R. T. & WARNKE, R. (1979) Non-Hodgkin's lymphoma: A study of the evolution of the disease based upon 92 autopsied cases. *Cancer*, **44**, 529.

ROBERTS, A. N., SMITH, K. L., DOWELL, B. L. & HUBBARD, A. K. (1978) Cultural, morphological, cell membrane, enzymatic and neoplastic properties of cell lines derived from a Hodgkin's disease lymph node. *Cancer Res.*, **38**, 3033.

ROBINOWITZ, B. N., NOGUCHI, S. & ROENICK, H. H. (1976) Tumor cell characterization in mycosis fungoides. *Cancer*, **37**, 1747.

SANDILANDS, G. P., GRAY, K., COONEY, A., BROWNING, J. D. & ANDERSON, J. R. (1975) Formation of auto-rosettes by peripheral blood lymphocytes. *Clin. Exp. Immunol.*, **22**, 493.

SCHEIN, P. S., CHABNER, B. A., CANELLOS, G. P., YOUNG, R. C., BERARD, C. W. & DEVITA, V. T. (1974) Potential for prolonged disease free survival following combination chemotherapy of non-Hodgkin's lymphoma. *Blood*, **43**, 181.

SCHEIN, P. S., DEVITA, V. T., HUBBARD, S. & 4 others (1976) Bleomycin, adriamycin, cyclophosphamide, vincristine, and prednisone (BACOP) combination chemotherapy in the treatment of advanced diffuse histiocytic lymphoma. *Ann. Intern. Med.*, **85**, 417.

SCHMITT, D., ALARIO, A., PERROT, H. & THIVOLET, J. (1977) Origin of Reed–Sternberg cell. *Lancet*, ii, 137.

SEN, L. & BORELLA, L. (1975) Clinical importance of lymphoblasts with T markers in childhood acute leukemia. *N. Engl. J. Med.*, **292**, 828.

STEIN, H. & MULLER-HERMELINK, H. K. (1977) Simultaneous presence of receptors for complement and sheep red blood cells on human fetal thymocytes. *Br. J. Haematol.*, **36**, 225.

STEIN, H., PETERSEN, N., GAEDICKE, G., LENNERT, K. & LANDBECK, G. (1976) Lymphoblastic lymphoma of convoluted or acid phosphatase type —A tumor of T precursor cells. *Int. J. Cancer*, **17**, 292.

STUART, A. E. WILLIAMS, A. R. W. & HABESHAW, J. A. (1977) Rosetting and other reactions of the Reed–Sternberg cell. *J. Pathol.* **122**, 81.

SUCHI, T., TAJIMA, K., NANBA, K. & 13 others (1979) Some problems on the histopathological diagnosis of non-Hodgkin's malignant lymphoma. A proposal of a new type. *Acta Pathol. Jap.*, **29**, 755.

TAYLOR, C. R. (1974) The nature of Reed–Sternberg cells and other malignant "reticulum" cells. *Lancet*, ii, 802.

TAYLOR, C. R. (1976) An immunohistological study of follicular lymphoma, reticulum cell sarcoma and Hodgkin's disease. *Eur. J. Cancer*, **12**, 61.

TONDER, O., MORSE, P. A. & HUMPHREY, L. J. (1974) Similarities of Fc receptors in human malignant tissue and normal lymphoid tissue. *J. Immunol.*, **113**, 1162.

TSUKIMOTO, I., WONG, K. Y. & LAMPKIN, B. C. (1976) Surface markers and prognostic factors in acute lymphoblastic leukemia. *N. Engl. J. Med.*, **294**, 245.

UCHIYAMA, T., YODIO, J., WAGAWA, K., TAKATSUKI, K. & UCHINO, H. (1977) Adult T-cell leukemia: Clinical and hematologic features of 16 cases. *Blood*, **50**, 481.

WALDRON, J. A., LEECH, J. H., GLICK, A. D., FLEXNER, J. M. & COLLINS, R. D. (1977) Malignant lymphoma of peripheral T-lymphocyte origin. Immunologic, pathologic, and clinical features in six patients. *Cancer*, **40**, 1604.

WEBSTER, R. (1961) Lymphosarcoma of the thymus: Its relation to acute lymphatic leukaemia. *Med. J. Aust.*, **48**, 582.

WEINER, M. S., BIANCO, C. & NUSSENZWEIG, V. (1973) Enhanced binding of neur-aminidase-treated sheep erythrocytes to human T lymphocytes. *Blood*, **42**, 939.

WEINSTEIN, H. J., VANCE, Z. B., JAFFE, N., BUELL, D., CASSADY, J. R. & NATHAN, D. G. (1979) Improved prognosis for patients with mediastinal lymphoblastic lymphoma. *Blood*, **53**, 687.

WODA, B. A. & KNOWLES, D. M. (1979) Nodular lymphocytic lymphoma eventuating into diffuse histiocytic lymphoma. Immunoperoxidase demonstration of monoclonality. *Cancer*, **43**, 303.

WYBRAN, J., CARR, M. C. & FUDENBERG, H. H. (1972) The human rosette-forming cell as a marker of a population of thymus-derived cells. *J. Clin. Invest.*, **51**, 2537.

YOUNG, R. C., CORDER, M. P., HAYNES, H. A. & DEVITA, V. T. (1973) Delayed hypersensitivity in Hodgkin's disease. A study of 103 untreated patients. *Am. J. Med.*, **52**, 63.

ZIEGLER, J. L. (1977) Treatment results of 54 American patients with Burkitt's lymphoma are similar to the African experience. *N. Engl. J. Med.*, **297**, 75.